# Compensating for geographic variation in detection probability with water depth improves abundance estimates of coastal marine megafauna

**DOI:** 10.1371/journal.pone.0191476

**Published:** 2018-01-25

**Authors:** Rie Hagihara, Rhondda E. Jones, Susan Sobtzick, Christophe Cleguer, Claire Garrigue, Helene Marsh

**Affiliations:** 1 College of Science and Engineering, James Cook University, Townsville, Queensland, Australia; 2 Centre for Tropical Water and Aquatic Ecosystem Research (TropWATER), James Cook University, Townsville, Queensland, Australia; 3 Murdoch University Cetacean Research Unit, School of Veterinary and Life Sciences, Murdoch University, South Street, Murdoch, Western Australia, Australia; 4 Opération Cétacés, Nouméa, New Calédonie; 5 Unité Mixte de Recherche (UMR) ENTROPIE (Institut de Recherche pour le Développement, Université de La Réunion, Centre National de la Recherche Scientifique), Laboratoire d’excellence LabEx, CORAIL, Nouméa, New Calédonie; Deakin University, AUSTRALIA

## Abstract

The probability of an aquatic animal being available for detection is typically <1. Accounting for covariates that reduce the probability of detection is important for obtaining robust estimates of the population abundance and determining its status and trends. The dugong (*Dugong dugon*) is a bottom-feeding marine mammal and a seagrass community specialist. We hypothesized that the probability of a dugong being available for detection is dependent on water depth and that dugongs spend more time underwater in deep-water seagrass habitats than in shallow-water seagrass habitats. We tested this hypothesis by quantifying the depth use of 28 wild dugongs fitted with GPS satellite transmitters and time-depth recorders (TDRs) at three sites with distinct seagrass depth distributions: 1) open waters supporting extensive seagrass meadows to 40 m deep (Torres Strait, 6 dugongs, 2015); 2) a protected bay (average water depth 6.8 m) with extensive shallow seagrass beds (Moreton Bay, 13 dugongs, 2011 and 2012); and 3) a mixture of lagoon, coral and seagrass habitats to 60 m deep (New Caledonia, 9 dugongs, 2013). The fitted instruments were used to measure the times the dugongs spent in the experimentally determined detection zones under various environmental conditions. The estimated probability of detection was applied to aerial survey data previously collected at each location. In general, dugongs were least available for detection in Torres Strait, and the population estimates increased 6–7 fold using depth-specific availability correction factors compared with earlier estimates that assumed homogeneous detection probability across water depth and location. Detection probabilities were higher in Moreton Bay and New Caledonia than Torres Strait because the water transparency in these two locations was much greater than in Torres Strait and the effect of correcting for depth-specific detection probability much less. The methodology has application to visual survey of coastal megafauna including surveys using Unmanned Aerial Vehicles.

## Introduction

A proportion of a target population is underwater during in-water surveys of aquatic wildlife and unavailable to visual observers. The probability of detecting an animal p(0, z) is often <1 (where z is the associated covariates, z = z_1_, z_2_, …, z_q_ [1]). The imperfect detection of the target species results in biased estimates of population size. In response to this problem, visual surveys are rigorously standardised to counter imperfect detection [1] and often use correction factors in an attempt to obtain absolute abundance estimates [1, 2]. Detection probability typically varies with survey conditions, circumstances, space and time [3, 4]. Studies of various species of marine megafauna (e.g., marine turtles [2, 5–7]; sharks [8]; marine mammals [9–12]) emphasise the importance of accounting for such heterogeneous detection probability to improve the accuracy of abundance estimates. When implemented in association with strategies to maximize precision, these approaches should improve the capacity of a series of surveys to determine population trends [13, 14], as required for the assessment of conservation status by the International Union for Conservation of Nature (IUCN) [15].

Three sources of bias in detection probability are recognised in past studies: absence [16], availability and perception [17]. Absence bias is caused by spatial or temporal shifts in the distribution of the target population with regard to a fixed survey area [16, 18, 19]. Availability bias occurs when animals are unavailable to be seen by observers because of unfavourable environmental conditions (e.g., sea state, turbidity, glare, water temperature, habitat type, tide and time of day) and animal traits (sex, group size, body size and colour, diving behaviour). Perception bias occurs when observers miss animals that are available for detection. Availability and perception biases can overlap, and past studies estimated these biases jointly or separately. In comparison with perception bias, availability bias can be substantial and spatially variable [1]. Thus the focus of this study is the availability bias.

Aerial surveys have been used extensively to estimate abundance and assess trends in populations of sirenians (manatees and dugongs [3, 12, 17, 20–22]). Sirenians are intermittently available to aerial observers and their availability is heterogeneous rather than static or discrete [4]. Craig and Reynolds (2004) assessed the population trends of the Florida manatee (*Trichechus manatus latirostris*) using a Bayesian approach to model combined availability and perception detection probabilities as functions of water depth and survey region. Edwards et al. (2007) obtained three separate (presence, availability and perception) site-specific probabilities for Florida manatees in warm-water winter refuges using radio-telemetry, marked individuals and time-depth recorders (TDRs). Fonnesbeck et al. (2009) extended this study and modelled availability detection probability as a function of air temperature and wind speed. The habitat-specific abundance was estimated by Langtimm et al. (2011) separately for offshore forage sites, inland fresh drinking waters and warm-water winter refuges.

For studies of dugongs, Pollock, Marsh [22] improved the technique developed by Marsh and Sinclair [17] and modelled availability detection probability external to the aerial survey using information on turbidity and sea state and the dive profiles of wild dugongs averaged over depth gradients. Population size was then estimated using the Horvitz-Thompson estimator [22]. Hagihara, Jones [23] extended Pollock’s study [19] and accounted for changes in diving and surfacing patterns with water depth over a limited range of bathymetric conditions.

The dugong is both an obligate air breather and a bottom feeder that primarily feeds on seagrass [24]. The diving patterns depend on the depth distribution of seagrass community, which in turn vary with bathymetry and environmental conditions [24]. The transit time between the surface and the sea floor increases with increasing depth of the seagrass community. We hypothesised that the probability of a dugong being available for detection varies across locations with different depth distributions of seagrass, which in turn are associated with different seagrass communities and dugong feeding modes [25, 26].

**Fig 1:**
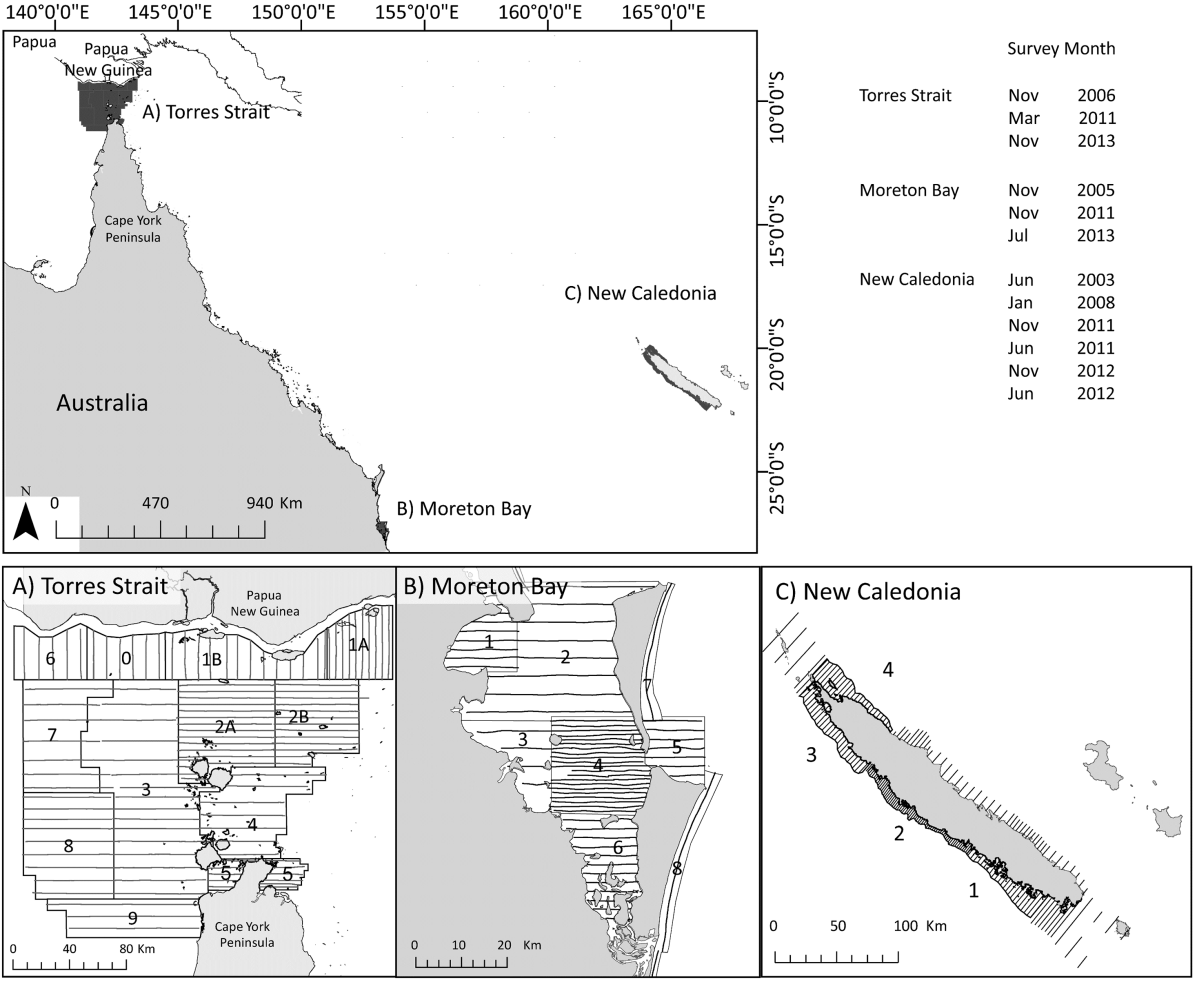
Maps showing dugong aerial survey blocks and transects in: A) Torres Strait, Australia, B) Moreton Bay, Australia and C) New Caledonia. Shaded areas and lines in the main map represent the survey area and transect lines. The month and year of the aerial surveys conducted at each location are shown in the top right corner.

To test this hypothesis, we examined the dive profiles of dugongs at three sites with different bio-physical features, depth distributions and seagrass communities (Fig 1): 1) oceanic waters dominated by deeper-water seagrass communities to 40 m (Torres Strait, Australia [27]); 2) a shallow protected bay (mean depth 6.8 m), characterised by large inter- and sub-tidal seagrass meadows (Moreton Bay, Australia [28]); and 3) coral and seagrass dominated lagoons with depths ranging from inter-tidal to 60 m (New Caledonia). We then used archival aerial survey data to compare aerial survey estimates of dugong abundance obtained using depth-corrected availability correction probabilities with the corresponding estimates obtained using the availability detection probabilities of Pollock et al. [18], who assumed that availability bias did not vary with water depth.

## Materials and methods

### Study sites

Torres Strait (9.50°S, 142.30°E) is formed by the inundated land bridge between the tip of Cape York Peninsula, Australia, and Papua New Guinea (Fig 1). The depth distribution of seagrass in Torres Strait extends to 40 m [29]; ~10,000 km^2^ of seagrass occurs in water >5 m deep [30]. Dugongs principally feed by cropping the leaves of dense seagrass meadows dominated by *Thallasia*, *Cymodocea* and *Syringodium* spp. [27]. These species represent a high proportion of the stomach contents recovered from dugongs in Torres Strait [26], which mainly feed in deeper-water seagrass meadows [27]. Moreton Bay (27.39°S, 153.32°E) is a large sub-tropical embayment. The bay supports seagrass meadows over shallow banks, typically <10 m deep [31]. Satellite-tracked dugongs frequently use these banks [32, 33] where dugongs spend ca. 40% of their time [34] excavating the above- and below-ground plants of pioneer seagrass such as *Halophila* spp. *and Halodule* spp. [28]. The main island of the New Caledonia archipelago (20.90°S, 165.62°E) located in eastern Melanesia is surrounded by a lagoon. The area where the dugongs were tracked (Cap Goulvain, Ouano and Noumea) consists of coral reefs, sandy bottoms and seagrass meadows [35, 36]. No information on dugong feeding modes or stomach contents is available from New Caledonia, but seagrass assemblages similar to Moreton Bay and Torres Strait have been recorded (*Thallasia*, *Cymodocea*, *Syringodium*, *Halophila* and *Halodule* spp. [37]).

### Availability detection probability

#### Depth of detection zones

Two pieces of information are required to estimate the availability detection probability of a dugong: 1) the depth range in which a dugong is visible to aerial observers, hereafter referred as the detection zone; and 2) the proportion of time a dugong is likely to be present in the detection zone. The range of each detection zone varies with the environmental conditions encountered during an aerial survey. We defined four levels of an Environmental Conditions Index (ECI) (Table 1, a composite index that incorporates various environmental conditions, especially turbidity and sea state). To determine the maximum depth of detection zones under various environmental conditions, we repeated the Dugong Secchi Disk experiment conducted by Pollock et al. [18] using TDRs with a finer depth resolution (± 0.08 m). The experiment was carried out between April 2013 and April 2014 on an opportunistic basis. The details of the experiment are described in S1 File.

**Table 1:** 

ECI	In-water visibility	Maximum depth (m) ± SE	Detection zone (m)
1	clear water, bottom clearly visible	All depths^1^	All depths^1^
2	variable clarity, bottom visible but not clearly	2.07 ± 0.50	0 to 2.0
3	clear water, bottom not visible	3.45 ± 0.59	0 to 3.5
4	turbid water, bottom not visible	1.59 ± 0.70	0 to 1.5

Means and standard errors (SE) of the maximum depths at which Dugong Secchi Disks were visible to experienced aerial observers under the Environmental Conditions Index (ECI), plus the depths of the detection zones used to estimate the proportion of time dugongs were available to aerial observers.

^1^ The experiment was not repeated for ECI1 because by definition all dugong models were available for detection by trained observers in aircraft at 500 ft (~152.4 m) under such environmental conditions.

#### Data collection and processing

We analysed data collected from 28 wild dugongs that were each fitted with GEN4 GPS/Argos Systems units (Telonics Inc., Mesa, Arizona, USA) and archival TDRs (Mk9 or MiniPAT; Wildlife Computers Woodinville, WA, USA) in October-November 2015 (Torres Strait), May-June 2011 [23] and July-September 2012 [33] (Moreton Bay) and September-October 2013 [38] (New Caledonia; Table 2). A GPS satellite transmitter was fitted to each dugong using a padded peduncle belt and a 3-m tether. A TDR was attached to the peduncle belt at the dugong’s tailstock and was thus unaffected by the length of the tether. This technique has been used successfully on more than 100 dugongs since the 1980s [33, 39]. Procedures for capturing, handling and attaching the tracking units followed Sheppard et al. [18] in Moreton Bay and New Caledonia or Fuentes, Cleguer [40] in Torres Strait. Details of individual dugongs are provided in S1 Table.

**Table 2:** 

Site	Year	Dugongs sampled*	TDR	Sampling interval (s)	Number of days with data (mean ± s.d.)
Torres Strait	2015	6 M	MiniPAT	75	4–60 ($\bar{\text{x}}=51\pm 21$)
Moreton Bay	2011	4 F	Mk9	1	16–78 ($\bar{\text{x}}=62\pm 31$)
Moreton Bay	2012	4 F; 5 M	Mk9	2	6–48 ($\bar{\text{x}}=30\pm 18$)
New Caledonia	2013	5 F; 4 M	Mk9	2	3–375 ($\bar{\text{x}}=52\pm 121$)

Summary of the dugongs studied, year of satellite tracking studies conducted, the equipment used and the number of days with both GPS and dive records used in the analysis.

*M = male; F = female

The GPS satellite transmitters were programmed to communicate with satellites every hour (25 animals) or 30 min (three animals deployed in Moreton Bay) (S1 Fig). The TDRs recorded depths every 1–2 s (Mk9) or 75 s (MiniPAT). Each MiniPAT was programmed to transmit the data to the satellites upon detachment after 60 days or upon earlier detachment. The Mk9 units were recovered for data retrieval. The GPS location data were retrieved from the Argos web site and decoded using the Telonics Data Converter; the Wildlife Computers portal was used to obtain the MiniPAT data. We used only high-quality location data: GPS fixes (± 2 to <10 m) and resolved Quick Fix Pseudoranging (QFP) fixes (± <75 m) for further analysis. The dive records from the Mk9 units were decoded by HexDecode. The sources of error in the depth records included: 1) drift in zero-reading; 2) wave action; 3) the depth resolution of the depth sensors; 4) the location of the depth sensors attached to the animals; and 5) the angle of animals’ body. Thus the depth records were zero-offset at each 10-min interval using custom software [41].

We assumed that the depth records collected by the Mk9 TDRs and MiniPATs were equivalent because: 1) both devices were made by the same manufacturer and had 0.5 m depth sensor resolution, 2) the accuracy of the depth records were tank-tested at a local 15m deep aquarium, and 3) the reliability of the depth profile provided by the maximum memory capacity of the MiniPATs (60 days) was confirmed by sub-sampling the depth records collected by the Mk9 TDRs assuming that the depth records were collected at the same frequency as the MiniPATSs (75sec) using the 1 sec Mk9 TDR sampling frequency as the standard. Some GPS transmitters and a MiniPAT were prematurely released from the dugongs due to unknown reasons or the malfunction of the weak link installed on the tracking apparatus. The weak link was designed to ensure the release of animals if the tether became entangled.

#### Statistical methods

Availability detection probabilities and their standard errors were estimated following Hagihara et al. [19]. The response was the proportion of time dugongs spent in each detection zone. Water depth and time of day were the two explanatory variables. The water depth was tidally adjusted at each location and time using a bathymetric model (± 100 m spatial resolution) [42]. The relationship between the response and water depth was non-monotonic and water depths were estimated from the model rather than measured empirically. Accordingly, water depth was divided into three empirically-selected depth categories: <5 m deep, 5 to <20 m deep and water ≥20 m deep. Time of day was divided into three equal periods: 0000-0800h, 0800-1600h and 1600-0000h. The 0800-1600h block reflected the time aerial surveys are conducted. We used Generalized Linear Mixed Models (GLMMs) assuming a binomial distribution and treated individual animals as a random variable. Autocorrelation between samples was minimal as each sample represented depth records from a 10-min block [41] and was collected at 1-h or 30-min interval. Statistical analysis was performed using lme4 (lme4_1.1–8) in R 3.1.3 (R Development Core Team, 2015).

### Abundance estimation

Population abundance was re-estimated using the archived aerial survey data collected in Torres Strait (2006, 2011 and 2013 all in summer), Moreton Bay (summer 2005, summer 2011 and winter 2013) and New Caledonia (winter 2003, summer 2008 [43]; and summers and winters 2011 and 2012 [38]). The aerial survey methodology is detailed in Marsh and Sinclair [17], Marsh and Sinclair [44], Pollock et al. [18] and S2 File for surveys in Australia and Garrigue, Patenaude [43] for surveys in New Caledonia.

## Results

### Availability detection probability

**Fig 2:**
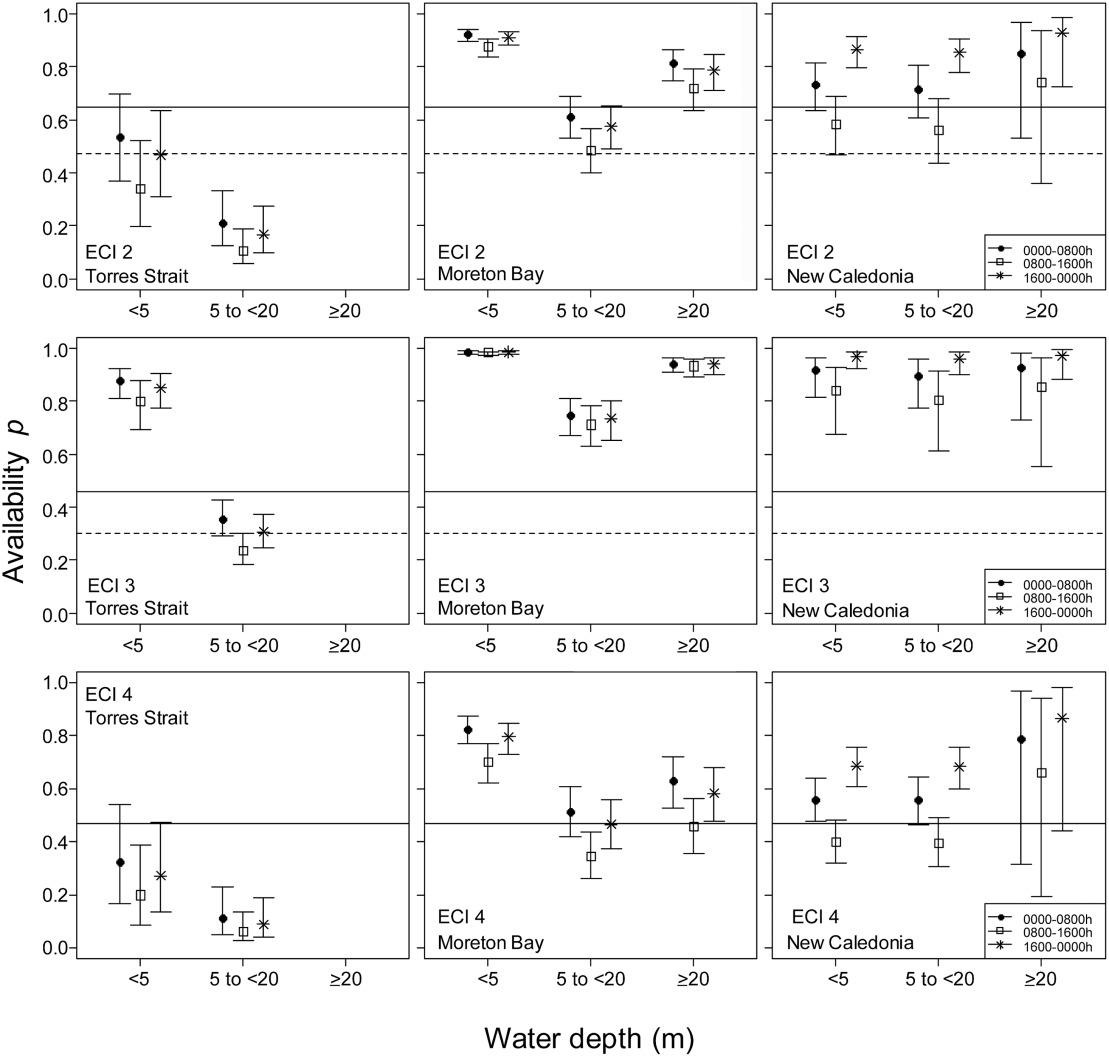
Availability detection probability estimates at the three locations, three time periods, three depth categories and three levels of environmental conditions index (ECI2-4). The horizontal lines represent the availability detection probabilities from Pollock et al. [18] for three turbidity levels and sea states (solid lines = optimal sea state; dotted lines = marginal sea state). Note in this study turbidity levels and sea state were merged to give a composite index ECI. The dotted lines in ECI4 are invisible as they overlap with the solid lines.

The time dugongs spent in each detection zone varied with location and time of day as well as water depth and environmental conditions. Dugongs in Torres Strait spent less time in the detection zones (Fig 2) than in Moreton Bay and New Caledonia, which were similar to each other. At all three locations, dugongs spent the smallest proportion of time in the detection zone during daylight hours (0800–1600 h). No satellite locations were obtained from dugongs in water >20m deep in Torres Strait. Therefore, the detection probability could not be estimated for that depth range at this location. This omission made only a trivial difference to the abundance estimates because few dugongs were sighted in this depth category during surveys (1% 2006, 4% 2011 and 3% 2013). The availability detection probabilities were the lowest in the intermediate depth category (5 to <20 m) in both Torres Strait and Moreton Bay but was no significant difference in New Caledonia between the shallowest (<5 m) and intermediate depth categories. S2 Table provides the model outputs.

### Abundance

**Fig 3:**
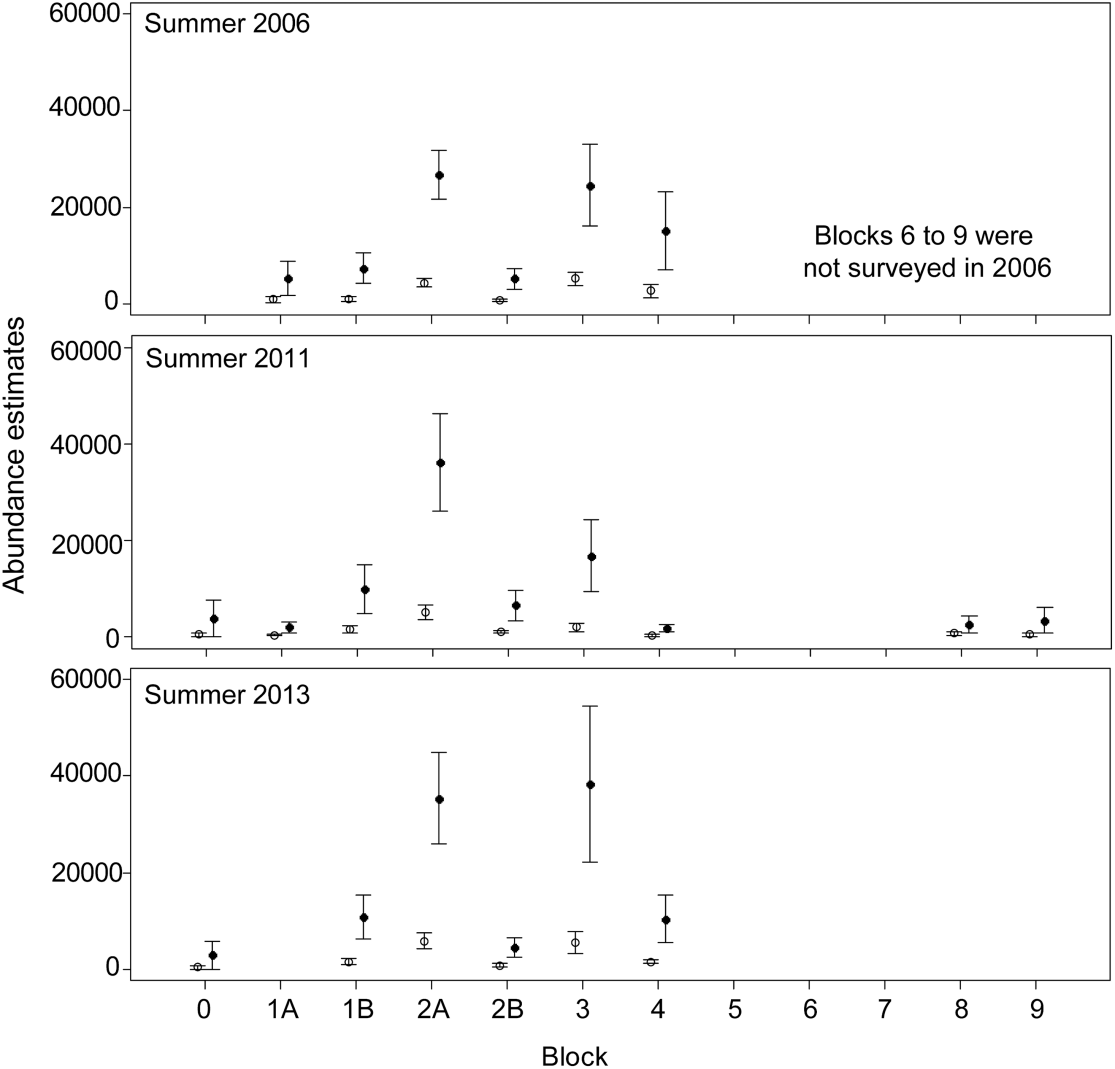
Population abundance estimates (± SE) (vertical lines) in Torres Strait obtained using the availability detection probabilities from Pollock et al. [18] (open circles) and this study (closed circles).

**Fig 4:**
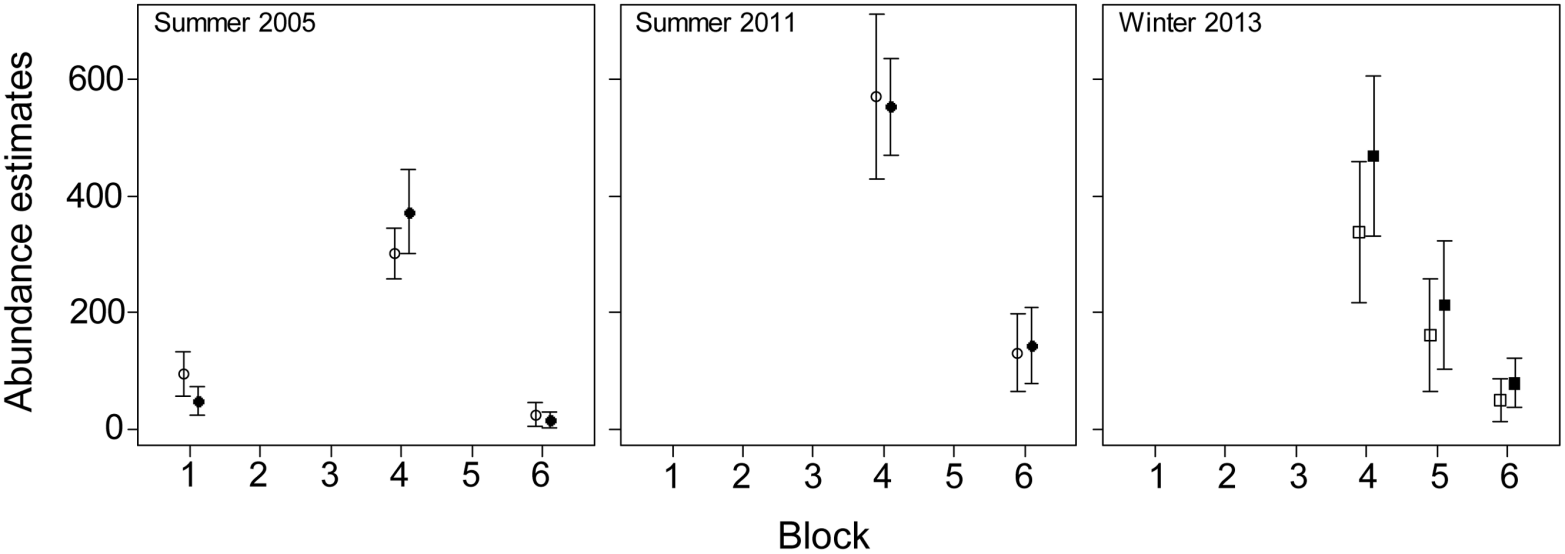
Population abundance estimates ± SE (vertical lines) in Moreton Bay obtained using the availability detection probabilities from Pollock et al. [18] (open squares) and this study (closed squares).

**Fig 5:**
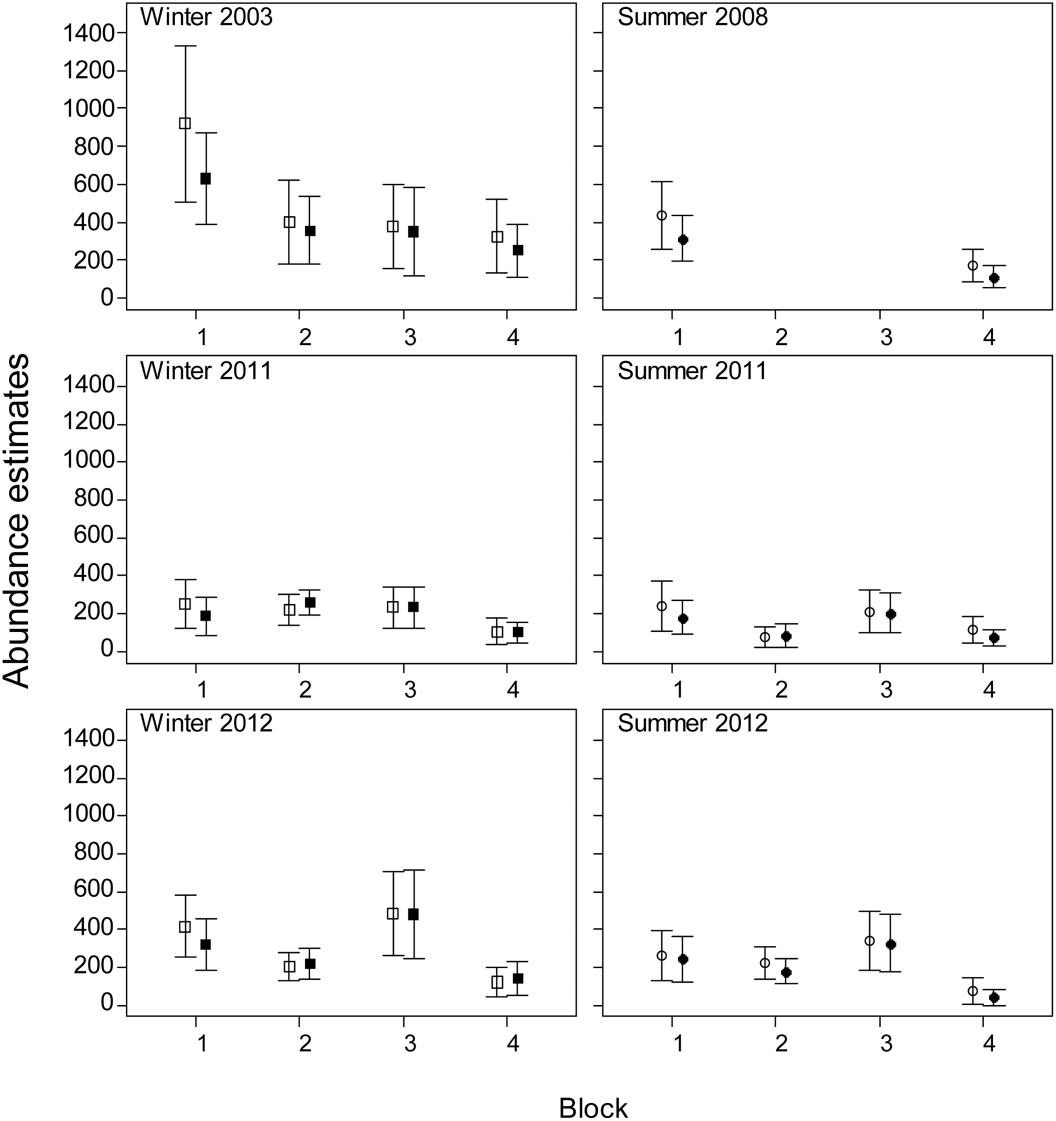
Population abundance estimates ± SE (vertical lines) in New Caledonia obtained using the availability detection probabilities from Pollock et al. [18] (open squares) and this study (closed squares).

The effect of using location specific correction factors on the estimates of dugong abundance was greatest for the Torres Strait survey region where the estimates were 5.7 to 6.6 times higher than the estimates using the detection probabilities from Pollock et al. [18] (Fig 3; Table 3). The survey blocks with the highest increase varied between surveys: 7.4 and 7.0 times higher in Blocks 1B and 2B in 2005, 8.1 times higher in Block 3 in 2011, and 7.4 times higher in Block 0 in 2013. In Moreton Bay, our estimates of total dugong abundance were very similar to the previous estimates except for the winter 2013 survey (Fig 4) when the estimates for all blocks were higher than for the earlier surveys (overall increase 1.28). In New Caledonia our abundance estimates were lower than the previous estimates for all surveys (Fig 5). The reduction was smaller in the winter surveys (3–22%) than in the summer surveys (12–30%). In all surveys, the coefficients of variation of the abundance estimates were very similar to those obtained using the previous methodology (Table 3; S3 Table).

**Table 3:** 

	Survey	Abundance ratio^a^	CV	
			1	2
Torres Strait	Summer 2006	5.71	0.16	0.16
	Summer 2011	6.61	0.17	0.18
	Summer 2013	6.52	0.19	0.20
Moreton Bay	Summer 2005	1.04	0.14	0.22
	Summer 2011	0.99	0.22	0.15
	Winter 2013	1.38	0.29	0.24
New Caledonia	Winter 2003	0.78	0.27	0.26
	Summer 2008	0.70	0.33	0.31
	Winter 2011	0.97	0.25	0.22
	Summer 2011	0.84	0.30	0.29
	Winter 2012	0.95	0.24	0.25
	Summer 2012	0.88	0.26	0.27

Ratio of the dugong abundance estimates using the availability detection probabilities from: 1) Pollock et al. [18] and 2) this study and the corresponding coefficients of variation (CV) of the abundance estimates.

^a^Abundance estimate from this study (numerator)/ corresponding estimate from Pollock et al. (2006) (denominator).

## Discussion

The proportion of time the tracked dugongs were close enough to the surface to be seen by aerial observers varied among locations with different bathymetries; the difference between Torres Strait and Moreton Bay/New Caledonia was substantial. These results are consistent with our hypothesis that the probability of a dugong being available for detection varies across locations with different depth distributions of seagrass communities. Dugongs in each location forage different assemblages of seagrass, and the feeding mode used may also be different (cropping is likely to be more frequently used by dugongs in Torres Strait whereas excavating may be more prevalent in dugongs found in Moreton Bay). This difference in seagrass community targeted by dugongs and feeding mode may lead to different proportions of time spent in the detection zones. The magnitude of each of these factors to availability bias is unknown but is a potential area of further investigation.

Although the dive profiles of the four dugongs tracked in Moreton Bay in 2011 showed that they transited rapidly between the surface and the seafloor in benthic dives and spent longer time in the bottom phase of a dive [45], the shallow bathymetric characteristics of this area presumably result in dugongs spending a longer proportion of their time in the detection zones. Nonetheless when in deeper waters, the tracked dugongs spent 72% of their time in the detection zone during the day. This result suggests that in Moreton Bay feeding is not the main activity for dugongs in deep water where seagrass tends to be sparse or absent. In addition, the energy expended by a diving dugong presumably increases with water depth.

In New Caledonia, the times dugongs spent in the detection zones were generally similar to those found in Moreton Bay except for small differences, especially in the shallowest depth category where the animals spent less time in the detection zone than in Moreton Bay. New Caledonian waters comprise a mixture of habitat types (coral reefs, seagrass meadows and sandy bottoms [37]) and although seagrass density is spatially variable most of seagrass meadows occur in water <5 m deep ([36], Claude Payri pers. comm.). These dugongs in New Caledonia may forage more frequently in this shallow depth range than in Moreton Bay where seagrass is available over vast banks extending to ca. 15 m deep. The longest times recorded for dugongs in the deepest depth category in New Caledonia are supported by observations of dugongs surface-resting at the edge of the lagoon [38].

### Abundance

The population size estimates in Torres Straits were 6–7 times higher in our study than estimated using Pollock et al.’s [18] availability detection probabilities. This result is largely explained by all of our detection probabilities for environmental conditions (ECI2-4) in the intermediate depth category (5 to <20 m) being lower than those of Pollock et al. [18] and the high proportion of dugong sightings in that depth category (66% of animals sighted in 2006, 60% in 2011 and 78% in 2013; Fig 2). Our larger population size estimates are consistent with Marsh et al. [46], who used several lines of evidence to demonstrate that the Torres Strait dugong population has been stable for at least 30 years despite high levels of indigenous harvest and concluded that the population size must be much larger than previously estimated.

The abundance estimates in Moreton Bay were similar to the previous estimates because most animals (73% in 2005 and 64% in 2011) were sighted in clear shallow water (EC1) and no correction was applicable to these sightings. In contrast, the abundance estimate for the 2013 winter survey was 1.28 times higher than the previous estimate. All our abundance estimates in New Caledonia were lower than the previous estimates because a large proportion of dugongs was sighted in environmental conditions ECI3 (46% 2003, 60% 2008, 41% and 38% in winter and summer 2011, 23% and 41% in winter and summer 2012) and the associated availability detection probabilities were higher than those from Pollock et al. [18]. The reduction in the abundance estimates was, however, smaller in the winter surveys (22% in 2003, 3% in 2011 and 5% in 2012) than for the summer surveys (30% in 2008, 16% in 2011 and 12% in 2012). Seasonal differences in the dugong’s diving and surfacing patterns may explain this difference.

Dugongs have limited thermal tolerance [47]. Some animals spend time in warmer oceanic waters during the winter months at locations such as Moreton Bay and New Caledonia at the high latitude limits to their range [32, 38]. They may spend more time near the surface during winter [38]. All extant sirenians have limited capacity to deal with heat loss [48] and there is increasing evidence of behavioural themo-regulation [24]. For example, Florida manatees surface-rest to absorb solar radiation after cold fronts and are also more easily seen in winter [49]. Further examination of the seasonal effects on the availability of dugongs, especially at the high latitude limits to their range is warranted.

## Conclusions

Our study demonstrates the importance of investigating the sources of and correcting for heterogeneous availability bias to improve the accuracy of abundance estimates when absolute estimates are required. Even when interest focusses on trends in density or abundance, it is important to investigate whether and how much p(0, z) varies to avoid the confounding effects of heterogeneous bias [1] on the index of interest. The approach used here could be customised for coastal marine wildlife such as marine turtles [6], dolphins and sharks and other visual techniques such as the use of Unmanned Aerial Vehicles [7, 50].

## Supporting information

S1 File Methods used for the Dugong Secchi Disk experiment. (DOCX)

S2 File Dugong aerial survey methodology. (DOCX)

S1 Fig GPS satellite points. (DOCX)

S1 Table Details of dugongs and fitted instruments. (DOCX)

S2 Table GLMM outputs. (DOCX)

S3 Table Abundance estimates. (DOCX)

S4 Table Data to reproduce availability bias estimates for Torres Strait. (DOCX)
