# Supplementary material for: Compensating for geographic variation in detection probability with water depth improves abundance estimates of coastal marine megafauna
Source: PLoS One. 2018 Jan 25;13(1):e0191476. doi: 10.1371/journal.pone.0191476 (PMC5784948; doi:10.1371/journal.pone.0191476)
Supplement: S1 File — (DOCX) [file pone.0191476.s001.docx]

# **S1 File. Methods used for the Dugong Secchi Disk experiment**

Estimating availability bias requires independent measurements external to an aerial survey of: 1) the depth range below the water surface in which a dugong is visible to aerial observers (Detection Zone); and 2) the proportion of time a dugong is likely to be present in the Detection Zone. This supplementary material describes the methodology to estimate the depths of Detection Zones for various environmental conditions.

Following Pollock et al. [[1](#_ENREF_1)], Dugong Secchi Disks – two-dimensional dugong replicas that mimicked the silhouette of a dugong as seen from an aerial survey aircraft – were used in experiments under a range of environmental conditions.

## **Dugong Secchi Disk.**

Two Dugong Secchi Disks were fabricated from marine plywood and fibreglass: one measured 2 m long; the second 2.4 m long. The dorsal surface of each Dugong Secchi Disk was painted to mimic wild dugongs as seen by aerial observers. A time-depth recorder (TDR) with improved depth resolution (0.08 m, DST milli-F manufactured by Star-Oddi, Gardabaer, Iceland) relative to the instruments used by Pollock et al. [1] was set to record depth at every second and mounted on each Dugong Secchi Disk. All TDRs were synchronised to the time of the GPS units carried by aerial observers. The buoyancy of the Dugong Secchi Disks was experimentally adjusted by attaching scuba weights, enabling them to be raised slowly from the sea floor. Each disk was attached to a separate pulley system.

## **Experimental design**

Before each experimental trial, both Dugong Secchi Disks were lowered to depths where they could not be seen by the two highly experienced aerial observers situated in a R44 helicopter hovering *ca*. 500 feet (normal dugong aerial survey height) above sea level. On receipt of radioed instructions from the lead observer, a vessel-based operator began raising the two disks in staggered random order. Each observer independently recorded the GPS time when the Dugong Secchi Disk was clearly sighted. The two observers were acoustically isolated during the experiment and did not communicate. The trial was repeated at least four times for each of the six combinations of ECI (3 levels) and Beaufort sea state (2 levels).

## **Estimating Detection Zones**

The experiment was carried out between April 2013 and April 2014 on an opportunistic basis. Conducting the experiment under the specified combinations of environmental conditions in uncontrollable field settings proved extremely difficult. It was impossible to standardise the environmental conditions (e.g., cloud cover, cloud shadow, glitter at the water surface, angle of sun), especially as some trials were necessarily conducted at different locations. The effect of sea state could not be separated from the other environmental variables that influence availability bias. Accordingly, we developed a composite index of the conditions that affect the capacity of observers to sight dugongs (Table 1). Level I of this Environmental Conditions Index occurs when the water is shallow and the seafloor clearly visible and by definition a dugong is available for detection, a situation which did not need to be experimentally verified.
