# Supplementary material for: Compensating for geographic variation in detection probability with water depth improves abundance estimates of coastal marine megafauna
Source: PLoS One. 2018 Jan 25;13(1):e0191476. doi: 10.1371/journal.pone.0191476 (PMC5784948; doi:10.1371/journal.pone.0191476)
Supplement: S2 File — (DOCX) [file pone.0191476.s002.docx]

# **S2 File. Dugong aerial survey methodology**

The standard dugong aerial survey methodology followed Marsh and Sinclair [[1](#_ENREF_1), [2](#_ENREF_2)] and Pollock et al. [[3](#_ENREF_3)] who describe the methodology in detail. All surveys in Australia were conducted using a 6-seater Partenavia 68B. Flight height in 2005 and 2006 was 137 m (450 ft); 152 m (500 ft) in later surveys, a permit requirement. In New Caledonia, the only available aircraft was a single engine Cessna. This aircraft flew at a height of ca. 900 feet (274 m) above sea level for safety reasons. The experimental work of Marsh and Sinclair [1] indicates that these differences in survey height should not make a substantive difference to the capacity of observers to detect dugongs. All aircraft were flown as close as possible to a ground speed of 100 knots.

The trained tandem team of two observers scanned the strip transect demarcated using fiberglass rods on their side of the aircraft. Transect widths were 200 m on either side of the aircraft for Australian surveys and 400 m wide each for surveys in New Caledonia. Distance categories (low=50 m, medium=100 m, high=150 m, and very high 150-200 m) within the strip were marked by colour bands on actual (Cessna) or artificial wing struts (Partenavia). Pollock et al. [3] found inconsistencies in the assignment of each sighting to the distance categories by mid and rear seat observers on the same side of the aircraft. As the number of dugong sightings did not differ among the distance categories, we followed Pollock et al. [3] and did not use the distance categories in estimating availability bias.

In Australia, a tandem team of two observers sitting in the middle and rear seats on each side of the aircraft was visually and acoustically isolated and reported their sightings into separate tracks of an audio recorder. The distance categories of each sighting within the strip enabled the survey team to decide if simultaneous sightings by tandem team members were of the same group of animals when reviewing the recordings after each day’s survey. This information was used to estimate perception bias. As explained by Pollock et al. [3], we found no decline in detection with distance across the strip, presumably because of the relatively narrow strip width. However, inter-observer comparisons indicated measurement error in the assignment of sightings to distance classes within the transect strip. Dugongs surface cryptically and for only 1-2 s [[4](#_ENREF_4), [5](#_ENREF_5)]. The cryptic nature of dugong surfacing and the often high sighting rate of the dugong meant that observers could not afford to take their eyes off the water to read an inclinometer. Thus following Pollock et al. [3], we did not to use distance category as a covariate in the analysis.

The surveys were conducted in passing mode. For each sighting, the observers recorded the total number of animals seen, number at the surface of the water, position in the transect sub-strip (e.g*.,* low or medium). The number of dugong calves (animals less than 2/3 of the size of the adult dugong and swimming in close proximity) was also recorded. On occasions when a group of dugongs encountered was too large to count in passing mode (>10 animals), the aircraft discontinued flying the transect and went into a circling mode in an effort to obtain a total count of the group before resuming the transect.

The survey leader seated next to the pilot collected data on environmental conditions at the beginning of each flight (cloud cover, cloud height, wind speed and direction, and air visibility) and each transect (cloud cover). There was a strict ceiling on weather conditions: no precipitation and Beaufort sea state <3. Every few minutes during each transect, and whenever conditions changed, the survey leader recorded sea state, water transparency, and glare (none; 0 to <25% of field of view affected; 25 <50% affected, >50% affected) on each side of the aircraft (the glare was assessed by the mid-seat observers).

**References**

1. Marsh H, Sinclair DF. An experimental evaluation of dugong and sea turtle aerial survey techniques. Aust Wildlife Res. 1989;16(6):639-50. PubMed PMID: WOS:A1989CU27100007.

2. Marsh H, Sinclair DF. Correcting for visibility bias in strip transect aerial surveys of aquatic fauna. J Wildlife Manage. 1989;53(4):1017-24. doi: Doi 10.2307/3809604. PubMed PMID: WOS:A1989CD23400027.

3. Pollock KH, Marsh HD, Lawler IR, Alldredge MW. Estimating animal abundance in heterogeneous environments: An application to aerial surveys for dugongs. J Wildlife Manage. 2006;70(1):255-62. doi: Doi 10.2193/0022-541x(2006)70[255:Eaaihe]2.0.Co;2. PubMed PMID: WOS:000237217900029.

4. Anderson PK, Birtles A. Behavior and ecology of dugong, *Dugong dugon* (Sirenia) - observations in Shoalwater and Cleveland Bays, Queensland. Aust Wildlife Res. 1978;5(1):1-23. PubMed PMID: WOS:A1978ER50100001.

5. Chilvers BL, Delean S, Gales NJ, Holley DK, Lawler IR, Marsh H, et al. Diving behaviour of dugongs, *Dugong dugon*. J Exp Mar Biol Ecol. 2004;304(2):203-24. doi: 10.1016/j.jembe.2003.12.010. PubMed PMID: WOS:000221572400004.
