# Supplementary material for: Compensating for geographic variation in detection probability with water depth improves abundance estimates of coastal marine megafauna
Source: PLoS One. 2018 Jan 25;13(1):e0191476. doi: 10.1371/journal.pone.0191476 (PMC5784948; doi:10.1371/journal.pone.0191476)

# **S1 Fig. GPS satellite points**

GPS satellite points from dugong tracking studies conducted in Torres Strait, Moreton Bay (Hagihara et al. 2014, Zeh et al. 2015) and New Caledonia (Cleguer 2015).


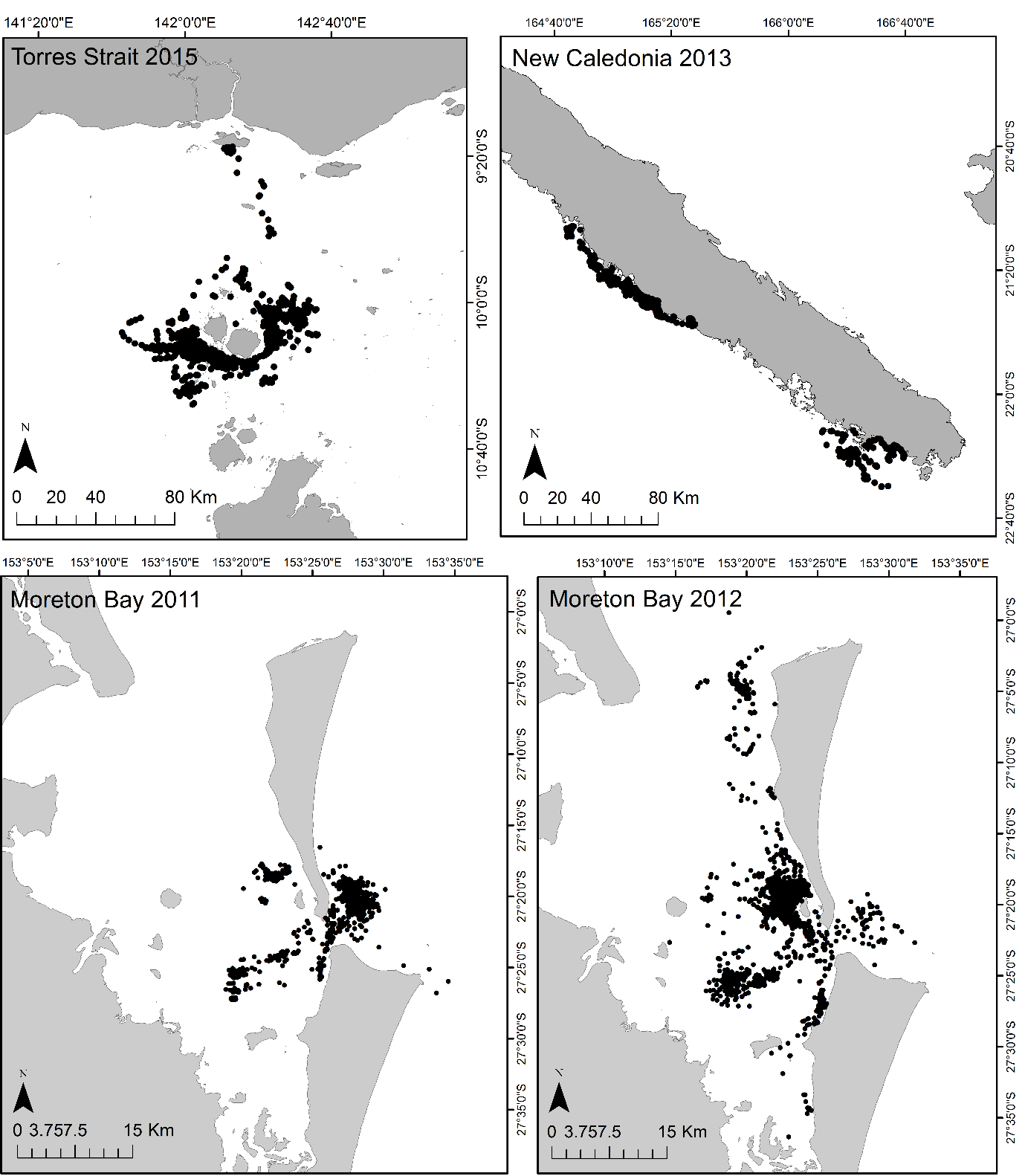

Supplement: S1 Fig — (DOCX) [file pone.0191476.s003.docx]
