# Supplementary material for: Compensating for geographic variation in detection probability with water depth improves abundance estimates of coastal marine megafauna
Source: PLoS One. 2018 Jan 25;13(1):e0191476. doi: 10.1371/journal.pone.0191476 (PMC5784948; doi:10.1371/journal.pone.0191476)
Supplement: S1 Table — (DOCX) [file pone.0191476.s004.docx]

# **S1 Table. Details of dugongs and fitted instruments**

|  | Year | Sex | Body length (cm) | Satellite tag sampling interval | TDR | TDR sampling interval (s) | # of days with both satellite and dive data | Data source |
| --- | --- | --- | --- | --- | --- | --- | --- | --- |
| Torres Strait | 2015 | M | 210 | 1 h | MiniPAT | 75 | 60 | This study |
|  |  | M | 240 | 1 h | MiniPAT | 75 | 60 |  |
|  |  | M | 270 | 1 h | MiniPAT | 75 | 4 |  |
|  |  | M | 240 | 1 h | MiniPAT | 75 | 60 |  |
|  |  | M | 210 | 1 h | MiniPAT | 75 | 60 |  |
|  |  | M | 270 | 1 h | MiniPAT | 75 | 60 |  |
| Moreton Bay | 2011 | F | 280 | 1 h | Mk9 | 1 | 78 | Hagihara et al. (2014) |
|  |  | F | 290 | 1 h | Mk9 | 1 | 77 |  |
|  |  | F | 290 | 1 h | Mk9 | 1 | 76 |  |
|  |  | F | 270 | 1 h | Mk9 | 1 | 16 |  |
|  | 2012 | M | 250 | 1 h | Mk9 | 2 | 19 | Zeh et al. (2015) |
|  |  | M | 300 | 1 h | Mk9 | 2 | 22 |  |
|  |  | F | 310 | 30 min | Mk9 | 2 | 27 |  |
|  |  | F | 220 | 30 min | Mk9 | 2 | 24 |  |
|  |  | F | 240 | 1 h | Mk9 | 2 | 28 |  |
|  |  | F | 210 | 1 h | Mk9 | 2 | 48 |  |
|  |  | M | 200 | 1 h | Mk9 | 2 | 70 |  |
|  |  | M | 290 | 30 min | Mk9 | 2 | 29 |  |
|  |  | M | 250 | 1 h | Mk9 | 2 | 6 |  |
| New Caledonia | 2013 | M | 247 | 1 h | Mk9 | 2 | 3 | Cleguer (2015) |
|  |  | F | 277 | 1 h | Mk9 | 2 | 13 |  |
|  |  | F | 271 | 1 h | Mk9 | 2 | 12 |  |
|  |  | F | 290 | 1 h | Mk9 | 2 | 7 |  |
|  |  | F | 227 | 1 h | Mk9 | 2 | 19 |  |
|  |  | M | 215 | 1 h | Mk9 | 2 | 16 |  |
|  |  | M | 240 | 1 h | Mk9 | 2 | 15 |  |
|  |  | F | 269 | 1 h | Mk9 | 2 | 5 |  |
|  |  | M | 226 | 1 h | Mk9 | 2 | 375 |  |
