# Supplementary material for: Compensating for geographic variation in detection probability with water depth improves abundance estimates of coastal marine megafauna
Source: PLoS One. 2018 Jan 25;13(1):e0191476. doi: 10.1371/journal.pone.0191476 (PMC5784948; doi:10.1371/journal.pone.0191476)
Supplement: S2 Table — (DOCX) [file pone.0191476.s005.docx]

# **S2 Table. GLMM outputs**

Coefficients for availability detection probability estimated for: A) Torres Strait, B) Moreton Bay and C) New Caledonia for Environmental Conditions Index (ECI) 2 to 4. ECI1 represents shallow clear water where the seafloor is clearly visible and by definition a dugong is available for detection. Thus the availability detection probability for ECI1 did not need to be experimentally verified.

|  | ECI | Variables | Estimate | Standard error | Z value | Pr(>\|z\|) |
| --- | --- | --- | --- | --- | --- | --- |
| A) Torres Strait | 2 | Intercept | 0.149 | 0.351 | 0.425 | 0.67 |
|  |  | Depth: 5 to <20 m | -1.480 | 0.202 | -7.328 | <0.001 |
|  |  | Time: 0800-1600h | -0.279 | 0.157 | -1.779 | 0.08 |
|  |  | Time: 1600-0000h | -0.816 | 0.201 | -4.063 | <0.001 |
|  | 3 | Intercept | 1.965 | 0.268 | 7.340 | <0.001 |
|  |  | Depth: 5 to <20 m | -2.561 | 0.247 | -10.371 | <0.001 |
|  |  | Time: 0800-1600h | -0.223 | 0.148 | -1.509 | 0.13 |
|  |  | Time: 1600-0000h | -0.573 | 0.169 | -3.381 | <0.001 |
|  | 4 | Intercept | -0.734 | 0.454 | -1.617 | 0.106 |
|  |  | Depth: 5 to <20 m | -1.332 | 0.216 | -6.169 | <0.001 |
|  |  | Time: 0800-1600h | -0.257 | 0.172 | -1.490 | 0.14 |
|  |  | Time: 1600-0000h | -0.666 | 0.220 | -3.027 | <0.01 |
| B) Moreton Bay | 2 | Intercept | 2.479 | 0.162 | 15.277 | <0.001 |
|  |  | Depth: 5 to <20 m | -2.018 | 0.087 | -23.186 | <0.001 |
|  |  | Depth: >20 m | -1.007 | 0.138 | -7.320 | <0.001 |
|  |  | Time: 0800-1600h | -0.531 | 0.074 | -7.207 | <0.001 |
|  |  | Time: 1600-0000h | -0.163 | 0.077 | -2.120 | <0.05 |
|  | 3 | Intercept | 4.242 | 0.198 | 21.421 | <0.001 |
|  |  | Depth: 5 to <20 m | -3.153 | 0.129 | -24.513 | <0.001 |
|  |  | Depth: >20 m | -1.429 | 0.228 | -6.257 | <0.001 |
|  |  | Time: 0800-1600h | -0.186 | 0.104 | -1.787 | 0.07 |
|  |  | Time: 1600-0000h | -0.070 | 0.111 | -0.628 | 0.53 |
|  | 4 | Intercept | 1.563 | 0.184 | 8.473 | <0.001 |
|  |  | Depth: 5 to <20 m | -1.502 | 0.080 | -18.790 | <0.001 |
|  |  | Depth: >20 m | -1.029 | 0.120 | -8.611 | <0.001 |
|  |  | Time: 0800-1600h | -0.706 | 0.064 | -11.120 | <0.001 |
|  |  | Time: 1600-0000h | -0.201 | 0.064 | -3.134 | <0.01 |
| C) New Caledonia | 2 | Intercept | 1.023 | 0.238 | 4.296 | <0.001 |
|  |  | Depth: 5 to <20 m | -0.097 | 0.128 | -0.760 | 0.45 |
|  |  | Depth: >20 m | 0.710 | 0.796 | 0.891 | 0.37 |
|  |  | Time: 0800-1600h | -0.686 | 0.117 | -5.866 | <0.001 |
|  |  | Time: 1600-0000h | 0.843 | 0.134 | 6.279 | <0.001 |
|  | 3 | Intercept | 2.412 | 0.471 | 5.124 | <0.001 |
|  |  | Depth: 5 to <20 m | -0.232 | 0.153 | -1.512 | 0.13 |
|  |  | Depth: >20 m | 0.116 | 0.641 | 0.181 | 0.86 |
|  |  | Time: 0800-1600h | -0.768 | 0.147 | -5.207 | <0.001 |
|  |  | Time: 1600-0000h | 1.012 | 0.188 | 5.386 | <0.001 |
|  | 4 | Intercept | 0.240 | 0.169 | 1.419 | 0.16 |
|  |  | Depth: 5 to <20 m | -0.011 | 0.112 | -0.099 | 0.92 |
|  |  | Depth: >20 m | 1.080 | 1.059 | 1.020 | 0.31 |
|  |  | Time: 0800-1600h | -0.653 | 0.106 | -6.171 | <0.001 |
|  |  | Time: 1600-0000h | 0.544 | 0.103 | 5.274 | <0.001 |
