# Supplementary material for: Compensating for geographic variation in detection probability with water depth improves abundance estimates of coastal marine megafauna
Source: PLoS One. 2018 Jan 25;13(1):e0191476. doi: 10.1371/journal.pone.0191476 (PMC5784948; doi:10.1371/journal.pone.0191476)
Supplement: S3 Table — (DOCX) [file pone.0191476.s006.docx]

# **S3 Table. Abundance estimates**

Estimates of dugong population abundance (+ SE) obtained using availability detection probabilities from Pollock et al. [[1](#_ENREF_1)] and this study.

|  | Survey | Population abundance (SE) | |
| --- | --- | --- | --- |
|  |  | Pollock | This study |
| Torres Strait | summer 2006 | 14,767 (2,292) | 84,389 (13,797) |
|  | summer 2011 | 12,603 (2,080) | 83,372 (14,693) |
|  | summer 2013 | 15,727 (2,942) | 102,519 (20,146) |
| Moreton Bay | summer 2005 | 422 (60) | 439 (97) |
|  | summer 2011 | 700 (156) | 696 (106) |
|  | winter 2013 | 551 (159) | 759 (181) |
| New Caledonia | winter 2003 | 2,026 (553) | 1,588 (407) |
|  | summer 2008 | 606 (200) | 426 (134) |
|  | winter 2011 | 810 (201) | 786 (171) |
|  | summer 2011 | 648 (196) | 545 (157) |
|  | winter 2012 | 1,227 (296) | 1,166 (293) |
|  | summer 2012 | 897 (231) | 792 (212) |

1. Pollock KH, Marsh HD, Lawler IR, Alldredge MW. Estimating animal abundance in heterogeneous environments: An application to aerial surveys for dugongs. J Wildlife Manage. 2006;70(1):255-62. doi: Doi 10.2193/0022-541x(2006)70[255:Eaaihe]2.0.Co;2. PubMed PMID: WOS:000237217900029.
