# Supplementary material for: Compensating for geographic variation in detection probability with water depth improves abundance estimates of coastal marine megafauna
Source: PLoS One. 2018 Jan 25;13(1):e0191476. doi: 10.1371/journal.pone.0191476 (PMC5784948; doi:10.1371/journal.pone.0191476)
Supplement: S4 Table — (DOCX) [file pone.0191476.s007.docx]

# **S4 Table. Data to reproduce availability bias estimates for Torres Strait**

## **ECI2**

| ID | waterDepth | timeCat | N | prop2 | Animal | depthCat |
| --- | --- | --- | --- | --- | --- | --- |
| 2 | 1 | 0.8h | 76 | 1 | D67389 | A.<5 |
| 4 | 1 | 16.0h | 8 | 1 | D67468 | A.<5 |
| 5 | 2 | 0.8h | 14 | 1 | D67468 | A.<5 |
| 6 | 2 | 0.8h | 154 | 0.948052 | D67389 | A.<5 |
| 7 | 2 | 0.8h | 16 | 0.1875 | D109776 | A.<5 |
| 8 | 2 | 0.8h | 16 | 0.25 | D34435 | A.<5 |
| 10 | 2 | 16.0h | 147 | 0.92517 | D67389 | A.<5 |
| 11 | 2 | 16.0h | 18 | 0.666667 | D67468 | A.<5 |
| 12 | 2 | 16.0h | 26 | 0.038462 | D34428 | A.<5 |
| 13 | 2 | 16.0h | 36 | 0.055556 | D109776 | A.<5 |
| 14 | 3 | 0.8h | 100 | 0.74 | D67389 | A.<5 |
| 15 | 3 | 0.8h | 121 | 0.140496 | D34435 | A.<5 |
| 16 | 3 | 0.8h | 16 | 0.6875 | D109776 | A.<5 |
| 17 | 3 | 0.8h | 18 | 0.111111 | D34428 | A.<5 |
| 18 | 3 | 16.0h | 161 | 0.807453 | D67389 | A.<5 |
| 19 | 3 | 16.0h | 34 | 0.823529 | D67468 | A.<5 |
| 20 | 3 | 16.0h | 40 | 0.125 | D109776 | A.<5 |
| 21 | 3 | 16.0h | 46 | 0.347826 | D34435 | A.<5 |
| 22 | 3 | 16.0h | 8 | 0 | D34428 | A.<5 |
| 23 | 4 | 0.8h | 16 | 0.375 | D109776 | A.<5 |
| 24 | 4 | 0.8h | 45 | 0.266667 | D34435 | A.<5 |
| 25 | 4 | 0.8h | 64 | 0.546875 | D67389 | A.<5 |
| 26 | 4 | 0.8h | 8 | 0 | D34428 | A.<5 |
| 27 | 4 | 16.0h | 10 | 0.1 | D34428 | A.<5 |
| 28 | 4 | 16.0h | 139 | 0.107914 | D34435 | A.<5 |
| 29 | 4 | 16.0h | 18 | 0.055556 | D109776 | A.<5 |
| 30 | 4 | 16.0h | 77 | 0.779221 | D67389 | A.<5 |
| 31 | 4 | 8.16h | 8 | 0 | D34428 | A.<5 |
| 32 | 4 | 8.16h | 8 | 0.125 | D67468 | A.<5 |
| 33 | 4 | 8.16h | 8 | 0.25 | D67389 | A.<5 |
| 34 | 5 | 0.8h | 16 | 0.125 | D109776 | B.5-<20 |
| 35 | 5 | 0.8h | 24 | 0.25 | D67389 | B.5-<20 |
| 36 | 5 | 0.8h | 42 | 0.071429 | D34435 | B.5-<20 |
| 38 | 5 | 0.8h | 8 | 0.25 | D34428 | B.5-<20 |
| 39 | 5 | 0.8h | 8 | 0.375 | D67468 | B.5-<20 |
| 40 | 5 | 16.0h | 14 | 0.214286 | D109776 | B.5-<20 |
| 41 | 5 | 16.0h | 39 | 0.538462 | D67389 | B.5-<20 |
| 42 | 5 | 16.0h | 8 | 0 | D34428 | B.5-<20 |
| 43 | 5 | 16.0h | 88 | 0.159091 | D34435 | B.5-<20 |
| 44 | 5 | 16.0h | 9 | 0.222222 | D34433 | B.5-<20 |
| 45 | 5 | 8.16h | 24 | 0.083333 | D34435 | B.5-<20 |
| 46 | 5 | 8.16h | 24 | 0.5 | D67389 | B.5-<20 |
| 47 | 5 | 8.16h | 8 | 0.25 | D109776 | B.5-<20 |
| 48 | 6 | 0.8h | 16 | 0.125 | D109776 | B.5-<20 |
| 49 | 6 | 0.8h | 16 | 0.125 | D67389 | B.5-<20 |
| 50 | 6 | 0.8h | 16 | 0.25 | D34428 | B.5-<20 |
| 51 | 6 | 0.8h | 8 | 0.125 | D34433 | B.5-<20 |
| 52 | 6 | 0.8h | 8 | 0.125 | D34435 | B.5-<20 |
| 53 | 6 | 0.8h | 8 | 0.125 | D67468 | B.5-<20 |
| 54 | 6 | 16.0h | 24 | 0.208333 | D34435 | B.5-<20 |
| 55 | 6 | 16.0h | 32 | 0.1875 | D34433 | B.5-<20 |
| 56 | 6 | 16.0h | 8 | 0 | D67389 | B.5-<20 |
| 57 | 6 | 8.16h | 22 | 0.090909 | D109776 | B.5-<20 |
| 58 | 6 | 8.16h | 24 | 0.291667 | D67389 | B.5-<20 |
| 59 | 6 | 8.16h | 40 | 0.05 | D34435 | B.5-<20 |
| 60 | 6 | 8.16h | 8 | 0.125 | D34428 | B.5-<20 |
| 61 | 6 | 8.16h | 8 | 0.25 | D34433 | B.5-<20 |
| 62 | 7 | 0.8h | 16 | 0.125 | D34433 | B.5-<20 |
| 63 | 7 | 0.8h | 16 | 0.25 | D67389 | B.5-<20 |
| 64 | 7 | 0.8h | 8 | 0 | D34428 | B.5-<20 |
| 65 | 7 | 0.8h | 8 | 0 | D34435 | B.5-<20 |
| 66 | 7 | 16.0h | 16 | 0.25 | D67389 | B.5-<20 |
| 67 | 7 | 16.0h | 48 | 0.166667 | D34433 | B.5-<20 |
| 68 | 7 | 16.0h | 64 | 0.171875 | D34435 | B.5-<20 |
| 69 | 7 | 16.0h | 8 | 0.25 | D34428 | B.5-<20 |
| 70 | 7 | 8.16h | 27 | 0.148148 | D34433 | B.5-<20 |
| 71 | 7 | 8.16h | 39 | 0.051282 | D34435 | B.5-<20 |
| 72 | 7 | 8.16h | 8 | 0.125 | D67389 | B.5-<20 |
| 73 | 8 | 0.8h | 40 | 0.25 | D34435 | B.5-<20 |
| 74 | 8 | 0.8h | 8 | 0.5 | D67389 | B.5-<20 |
| 75 | 8 | 0.8h | 9 | 0.111111 | D34428 | B.5-<20 |
| 76 | 8 | 16.0h | 16 | 0.375 | D67389 | B.5-<20 |
| 77 | 8 | 16.0h | 24 | 0.125 | D34435 | B.5-<20 |
| 78 | 8 | 16.0h | 25 | 0.12 | D109776 | B.5-<20 |
| 79 | 8 | 16.0h | 8 | 0 | D34428 | B.5-<20 |
| 80 | 8 | 8.16h | 14 | 0.142857 | D34435 | B.5-<20 |
| 81 | 8 | 8.16h | 8 | 0.375 | D34428 | B.5-<20 |
| 82 | 9 | 0.8h | 16 | 0.1875 | D109776 | B.5-<20 |
| 83 | 9 | 0.8h | 22 | 0.090909 | D34428 | B.5-<20 |
| 84 | 9 | 0.8h | 48 | 0.1875 | D34435 | B.5-<20 |
| 85 | 9 | 0.8h | 8 | 0.125 | D34433 | B.5-<20 |
| 86 | 9 | 0.8h | 8 | 0.625 | D67389 | B.5-<20 |
| 88 | 9 | 16.0h | 48 | 0.125 | D109776 | B.5-<20 |
| 89 | 9 | 16.0h | 56 | 0.196429 | D34435 | B.5-<20 |
| 90 | 9 | 16.0h | 8 | 0.5 | D67389 | B.5-<20 |
| 91 | 9 | 8.16h | 8 | 0 | D34435 | B.5-<20 |
| 92 | 9 | 8.16h | 8 | 0.125 | D109776 | B.5-<20 |
| 93 | 9 | 8.16h | 8 | 0.125 | D34428 | B.5-<20 |
| 95 | 10 | 0.8h | 10 | 0.1 | D109776 | B.5-<20 |
| 96 | 10 | 0.8h | 15 | 0 | D34428 | B.5-<20 |
| 97 | 10 | 0.8h | 24 | 0.041667 | D34435 | B.5-<20 |
| 98 | 10 | 0.8h | 24 | 0.25 | D67389 | B.5-<20 |
| 99 | 10 | 16.0h | 13 | 0.461538 | D67468 | B.5-<20 |
| 100 | 10 | 16.0h | 16 | 0.0625 | D34428 | B.5-<20 |
| 101 | 10 | 16.0h | 33 | 0.060606 | D109776 | B.5-<20 |
| 102 | 10 | 8.16h | 11 | 0.181818 | D67468 | B.5-<20 |
| 103 | 10 | 8.16h | 16 | 0.1875 | D34433 | B.5-<20 |
| 104 | 10 | 8.16h | 16 | 0.25 | D67389 | B.5-<20 |
| 105 | 10 | 8.16h | 24 | 0.125 | D34435 | B.5-<20 |
| 106 | 10 | 8.16h | 39 | 0.102564 | D34428 | B.5-<20 |
| 107 | 10 | 8.16h | 8 | 0.5 | D109776 | B.5-<20 |
| 108 | 11 | 0.8h | 134 | 0.231343 | D34433 | B.5-<20 |
| 109 | 11 | 0.8h | 24 | 0.041667 | D34435 | B.5-<20 |
| 110 | 11 | 0.8h | 40 | 0.175 | D34428 | B.5-<20 |
| 111 | 11 | 0.8h | 72 | 0.194444 | D109776 | B.5-<20 |
| 112 | 11 | 0.8h | 74 | 0.554054 | D67389 | B.5-<20 |
| 113 | 11 | 0.8h | 8 | 0.125 | D67468 | B.5-<20 |
| 114 | 11 | 16.0h | 16 | 0 | D34435 | B.5-<20 |
| 115 | 11 | 16.0h | 32 | 0.15625 | D34428 | B.5-<20 |
| 116 | 11 | 16.0h | 37 | 0.135135 | D34433 | B.5-<20 |
| 117 | 11 | 16.0h | 64 | 0.15625 | D67389 | B.5-<20 |
| 118 | 11 | 16.0h | 8 | 0.125 | D109776 | B.5-<20 |
| 119 | 11 | 8.16h | 16 | 0.125 | D67468 | B.5-<20 |
| 120 | 11 | 8.16h | 16 | 0.25 | D67389 | B.5-<20 |
| 121 | 11 | 8.16h | 40 | 0.2 | D34428 | B.5-<20 |
| 122 | 11 | 8.16h | 50 | 0.06 | D34433 | B.5-<20 |
| 123 | 11 | 8.16h | 8 | 0.125 | D109776 | B.5-<20 |
| 124 | 12 | 0.8h | 148 | 0.189189 | D34428 | B.5-<20 |
| 125 | 12 | 0.8h | 247 | 0.48583 | D67389 | B.5-<20 |
| 126 | 12 | 0.8h | 536 | 0.277985 | D34433 | B.5-<20 |
| 127 | 12 | 0.8h | 58 | 0.137931 | D34435 | B.5-<20 |
| 128 | 12 | 0.8h | 75 | 0.226667 | D109776 | B.5-<20 |
| 129 | 12 | 0.8h | 8 | 0.375 | D67468 | B.5-<20 |
| 130 | 12 | 16.0h | 119 | 0.109244 | D34435 | B.5-<20 |
| 131 | 12 | 16.0h | 16 | 0.3125 | D67468 | B.5-<20 |
| 132 | 12 | 16.0h | 24 | 0.083333 | D109776 | B.5-<20 |
| 133 | 12 | 16.0h | 371 | 0.196765 | D34433 | B.5-<20 |
| 134 | 12 | 16.0h | 57 | 0.175439 | D34428 | B.5-<20 |
| 135 | 12 | 16.0h | 72 | 0.152778 | D67389 | B.5-<20 |
| 136 | 12 | 8.16h | 301 | 0.136213 | D34433 | B.5-<20 |
| 137 | 12 | 8.16h | 31 | 0.16129 | D34435 | B.5-<20 |
| 138 | 12 | 8.16h | 37 | 0.378378 | D67389 | B.5-<20 |
| 139 | 12 | 8.16h | 40 | 0.175 | D34428 | B.5-<20 |
| 140 | 12 | 8.16h | 8 | 0.125 | D67468 | B.5-<20 |
| 141 | 12 | 8.16h | 8 | 0.25 | D109776 | B.5-<20 |
| 142 | 13 | 0.8h | 216 | 0.347222 | D67389 | B.5-<20 |
| 143 | 13 | 0.8h | 24 | 0.25 | D109776 | B.5-<20 |
| 144 | 13 | 0.8h | 243 | 0.238683 | D34433 | B.5-<20 |
| 145 | 13 | 0.8h | 62 | 0.193548 | D34428 | B.5-<20 |
| 146 | 13 | 0.8h | 8 | 0.125 | D34435 | B.5-<20 |
| 147 | 13 | 16.0h | 111 | 0.225225 | D34428 | B.5-<20 |
| 148 | 13 | 16.0h | 214 | 0.252336 | D67389 | B.5-<20 |
| 149 | 13 | 16.0h | 58 | 0.103448 | D34435 | B.5-<20 |
| 150 | 13 | 16.0h | 605 | 0.213223 | D34433 | B.5-<20 |
| 151 | 13 | 16.0h | 85 | 0.164706 | D109776 | B.5-<20 |
| 152 | 13 | 8.16h | 16 | 0.0625 | D109776 | B.5-<20 |
| 153 | 13 | 8.16h | 16 | 0.1875 | D67468 | B.5-<20 |
| 154 | 13 | 8.16h | 170 | 0.235294 | D67389 | B.5-<20 |
| 155 | 13 | 8.16h | 653 | 0.143951 | D34433 | B.5-<20 |
| 156 | 13 | 8.16h | 66 | 0.075758 | D34435 | B.5-<20 |
| 157 | 13 | 8.16h | 77 | 0.116883 | D34428 | B.5-<20 |
| 158 | 14 | 0.8h | 16 | 0.4375 | D109776 | B.5-<20 |
| 159 | 14 | 0.8h | 181 | 0.41989 | D67389 | B.5-<20 |
| 160 | 14 | 0.8h | 27 | 0.074074 | D34435 | B.5-<20 |
| 161 | 14 | 0.8h | 33 | 0.212121 | D34428 | B.5-<20 |
| 162 | 14 | 0.8h | 71 | 0.197183 | D34433 | B.5-<20 |
| 163 | 14 | 16.0h | 16 | 0.4375 | D67468 | B.5-<20 |
| 164 | 14 | 16.0h | 263 | 0.262357 | D67389 | B.5-<20 |
| 165 | 14 | 16.0h | 288 | 0.15625 | D34433 | B.5-<20 |
| 166 | 14 | 16.0h | 40 | 0.15 | D109776 | B.5-<20 |
| 167 | 14 | 16.0h | 56 | 0.142857 | D34428 | B.5-<20 |
| 168 | 14 | 16.0h | 8 | 0.375 | D34435 | B.5-<20 |
| 169 | 14 | 8.16h | 121 | 0.198347 | D67389 | B.5-<20 |
| 170 | 14 | 8.16h | 128 | 0.0625 | D34428 | B.5-<20 |
| 171 | 14 | 8.16h | 16 | 0 | D34435 | B.5-<20 |
| 172 | 14 | 8.16h | 273 | 0.142857 | D34433 | B.5-<20 |
| 173 | 14 | 8.16h | 49 | 0.204082 | D109776 | B.5-<20 |
| 174 | 15 | 0.8h | 16 | 0.125 | D34428 | B.5-<20 |
| 175 | 15 | 0.8h | 32 | 0.40625 | D67389 | B.5-<20 |
| 176 | 15 | 0.8h | 64 | 0.1875 | D34433 | B.5-<20 |
| 177 | 15 | 0.8h | 8 | 0.375 | D109776 | B.5-<20 |
| 178 | 15 | 16.0h | 43 | 0.348837 | D67389 | B.5-<20 |
| 179 | 15 | 16.0h | 46 | 0.086957 | D34428 | B.5-<20 |
| 180 | 15 | 8.16h | 52 | 0.134615 | D34433 | B.5-<20 |
| 181 | 15 | 8.16h | 56 | 0.196429 | D67389 | B.5-<20 |
| 182 | 15 | 8.16h | 64 | 0.09375 | D34428 | B.5-<20 |
| 183 | 15 | 8.16h | 8 | 0 | D34435 | B.5-<20 |
| 184 | 16 | 0.8h | 16 | 0.9375 | D67389 | B.5-<20 |
| 186 | 16 | 16.0h | 22 | 0.363636 | D67389 | B.5-<20 |
| 187 | 16 | 16.0h | 8 | 0 | D34428 | B.5-<20 |
| 188 | 16 | 8.16h | 24 | 0.125 | D34428 | B.5-<20 |
| 189 | 16 | 8.16h | 8 | 0.25 | D34433 | B.5-<20 |
| 190 | 17 | 0.8h | 8 | 0 | D34428 | B.5-<20 |
| 191 | 17 | 8.16h | 24 | 0.125 | D34433 | B.5-<20 |
| 192 | 17 | 8.16h | 8 | 0 | D34428 | B.5-<20 |
| 193 | 17 | 8.16h | 8 | 0.125 | D67389 | B.5-<20 |

## **ECI3**

| ID | waterDepth | timeCat | N | Animal | prop3.5 | depthCat |
| --- | --- | --- | --- | --- | --- | --- |
| 3 | 1 | 0.8h | 76 | D67389 | 1 | A.<5 |
| 4 | 1 | 16.0h | 8 | D67468 | 1 | A.<5 |
| 5 | 2 | 0.8h | 16 | D109776 | 0.8125 | A.<5 |
| 6 | 2 | 16.0h | 36 | D109776 | 1 | A.<5 |
| 8 | 2 | 16.0h | 26 | D34428 | 0.538462 | A.<5 |
| 9 | 2 | 0.8h | 16 | D34435 | 0.8125 | A.<5 |
| 10 | 2 | 0.8h | 154 | D67389 | 0.987013 | A.<5 |
| 11 | 2 | 16.0h | 147 | D67389 | 1 | A.<5 |
| 12 | 2 | 0.8h | 14 | D67468 | 1 | A.<5 |
| 13 | 2 | 16.0h | 18 | D67468 | 0.833333 | A.<5 |
| 14 | 3 | 0.8h | 16 | D109776 | 1 | A.<5 |
| 15 | 3 | 16.0h | 40 | D109776 | 0.975 | A.<5 |
| 16 | 3 | 0.8h | 18 | D34428 | 0.277778 | A.<5 |
| 17 | 3 | 16.0h | 8 | D34428 | 0.875 | A.<5 |
| 18 | 3 | 0.8h | 121 | D34435 | 0.933884 | A.<5 |
| 19 | 3 | 16.0h | 46 | D34435 | 0.978261 | A.<5 |
| 20 | 3 | 0.8h | 100 | D67389 | 0.81 | A.<5 |
| 21 | 3 | 16.0h | 161 | D67389 | 0.993789 | A.<5 |
| 22 | 3 | 16.0h | 34 | D67468 | 1 | A.<5 |
| 23 | 4 | 0.8h | 16 | D109776 | 0.5 | A.<5 |
| 24 | 4 | 16.0h | 18 | D109776 | 1 | A.<5 |
| 25 | 4 | 0.8h | 8 | D34428 | 0 | A.<5 |
| 26 | 4 | 16.0h | 10 | D34428 | 0.8 | A.<5 |
| 27 | 4 | 8.16h | 8 | D34428 | 0.125 | A.<5 |
| 28 | 4 | 0.8h | 45 | D34435 | 0.555556 | A.<5 |
| 29 | 4 | 16.0h | 139 | D34435 | 0.769784 | A.<5 |
| 30 | 4 | 0.8h | 64 | D67389 | 0.671875 | A.<5 |
| 31 | 4 | 16.0h | 77 | D67389 | 0.87013 | A.<5 |
| 32 | 4 | 8.16h | 8 | D67389 | 0.25 | A.<5 |
| 33 | 4 | 8.16h | 8 | D67468 | 0.125 | A.<5 |
| 34 | 5 | 0.8h | 16 | D109776 | 0.125 | B.5-<20 |
| 35 | 5 | 16.0h | 14 | D109776 | 0.571429 | B.5-<20 |
| 36 | 5 | 8.16h | 8 | D109776 | 0.375 | B.5-<20 |
| 37 | 5 | 0.8h | 8 | D34428 | 0.25 | B.5-<20 |
| 38 | 5 | 16.0h | 8 | D34428 | 0.125 | B.5-<20 |
| 40 | 5 | 16.0h | 9 | D34433 | 0.222222 | B.5-<20 |
| 41 | 5 | 0.8h | 42 | D34435 | 0.285714 | B.5-<20 |
| 42 | 5 | 16.0h | 88 | D34435 | 0.534091 | B.5-<20 |
| 43 | 5 | 8.16h | 24 | D34435 | 0.125 | B.5-<20 |
| 44 | 5 | 0.8h | 24 | D67389 | 0.458333 | B.5-<20 |
| 45 | 5 | 16.0h | 39 | D67389 | 0.589744 | B.5-<20 |
| 46 | 5 | 8.16h | 24 | D67389 | 0.5 | B.5-<20 |
| 47 | 5 | 0.8h | 8 | D67468 | 0.875 | B.5-<20 |
| 48 | 6 | 0.8h | 16 | D109776 | 0.125 | B.5-<20 |
| 49 | 6 | 8.16h | 22 | D109776 | 0.181818 | B.5-<20 |
| 50 | 6 | 0.8h | 16 | D34428 | 0.25 | B.5-<20 |
| 51 | 6 | 8.16h | 8 | D34428 | 0.375 | B.5-<20 |
| 52 | 6 | 0.8h | 8 | D34433 | 0.25 | B.5-<20 |
| 53 | 6 | 16.0h | 32 | D34433 | 0.40625 | B.5-<20 |
| 54 | 6 | 8.16h | 8 | D34433 | 0.25 | B.5-<20 |
| 55 | 6 | 0.8h | 8 | D34435 | 0.125 | B.5-<20 |
| 56 | 6 | 16.0h | 24 | D34435 | 0.5 | B.5-<20 |
| 57 | 6 | 8.16h | 40 | D34435 | 0.15 | B.5-<20 |
| 58 | 6 | 0.8h | 16 | D67389 | 0.125 | B.5-<20 |
| 59 | 6 | 16.0h | 8 | D67389 | 0 | B.5-<20 |
| 60 | 6 | 8.16h | 24 | D67389 | 0.291667 | B.5-<20 |
| 61 | 6 | 0.8h | 8 | D67468 | 0.375 | B.5-<20 |
| 62 | 7 | 0.8h | 8 | D34428 | 0.375 | B.5-<20 |
| 63 | 7 | 16.0h | 8 | D34428 | 0.25 | B.5-<20 |
| 64 | 7 | 0.8h | 16 | D34433 | 0.625 | B.5-<20 |
| 65 | 7 | 16.0h | 48 | D34433 | 0.229167 | B.5-<20 |
| 66 | 7 | 8.16h | 27 | D34433 | 0.185185 | B.5-<20 |
| 67 | 7 | 0.8h | 8 | D34435 | 0 | B.5-<20 |
| 68 | 7 | 16.0h | 64 | D34435 | 0.515625 | B.5-<20 |
| 69 | 7 | 8.16h | 39 | D34435 | 0.102564 | B.5-<20 |
| 70 | 7 | 0.8h | 16 | D67389 | 0.3125 | B.5-<20 |
| 71 | 7 | 16.0h | 16 | D67389 | 0.3125 | B.5-<20 |
| 72 | 7 | 8.16h | 8 | D67389 | 0.125 | B.5-<20 |
| 73 | 8 | 16.0h | 25 | D109776 | 0.24 | B.5-<20 |
| 74 | 8 | 0.8h | 9 | D34428 | 0.333333 | B.5-<20 |
| 75 | 8 | 16.0h | 8 | D34428 | 0.25 | B.5-<20 |
| 76 | 8 | 8.16h | 8 | D34428 | 0.625 | B.5-<20 |
| 77 | 8 | 0.8h | 40 | D34435 | 0.825 | B.5-<20 |
| 78 | 8 | 16.0h | 24 | D34435 | 0.708333 | B.5-<20 |
| 79 | 8 | 8.16h | 14 | D34435 | 0.214286 | B.5-<20 |
| 80 | 8 | 0.8h | 8 | D67389 | 0.5 | B.5-<20 |
| 81 | 8 | 16.0h | 16 | D67389 | 0.5625 | B.5-<20 |
| 82 | 9 | 0.8h | 16 | D109776 | 0.25 | B.5-<20 |
| 83 | 9 | 16.0h | 48 | D109776 | 0.1875 | B.5-<20 |
| 84 | 9 | 8.16h | 8 | D109776 | 0.125 | B.5-<20 |
| 85 | 9 | 0.8h | 22 | D34428 | 0.227273 | B.5-<20 |
| 87 | 9 | 8.16h | 8 | D34428 | 0.125 | B.5-<20 |
| 88 | 9 | 0.8h | 8 | D34433 | 0.75 | B.5-<20 |
| 89 | 9 | 0.8h | 48 | D34435 | 0.416667 | B.5-<20 |
| 90 | 9 | 16.0h | 56 | D34435 | 0.714286 | B.5-<20 |
| 91 | 9 | 8.16h | 8 | D34435 | 0.125 | B.5-<20 |
| 92 | 9 | 0.8h | 8 | D67389 | 0.625 | B.5-<20 |
| 93 | 9 | 16.0h | 8 | D67389 | 1 | B.5-<20 |
| 94 | 10 | 0.8h | 10 | D109776 | 0.2 | B.5-<20 |
| 95 | 10 | 16.0h | 33 | D109776 | 0.090909 | B.5-<20 |
| 96 | 10 | 8.16h | 8 | D109776 | 0.75 | B.5-<20 |
| 97 | 10 | 0.8h | 15 | D34428 | 0.266667 | B.5-<20 |
| 98 | 10 | 16.0h | 16 | D34428 | 0.3125 | B.5-<20 |
| 99 | 10 | 8.16h | 39 | D34428 | 0.153846 | B.5-<20 |
| 101 | 10 | 8.16h | 16 | D34433 | 0.1875 | B.5-<20 |
| 102 | 10 | 0.8h | 24 | D34435 | 0.083333 | B.5-<20 |
| 103 | 10 | 8.16h | 24 | D34435 | 0.125 | B.5-<20 |
| 104 | 10 | 0.8h | 24 | D67389 | 0.333333 | B.5-<20 |
| 105 | 10 | 8.16h | 16 | D67389 | 0.3125 | B.5-<20 |
| 106 | 10 | 16.0h | 13 | D67468 | 0.461538 | B.5-<20 |
| 107 | 10 | 8.16h | 11 | D67468 | 0.272727 | B.5-<20 |
| 108 | 11 | 0.8h | 72 | D109776 | 0.347222 | B.5-<20 |
| 109 | 11 | 16.0h | 8 | D109776 | 0.125 | B.5-<20 |
| 110 | 11 | 8.16h | 8 | D109776 | 0.125 | B.5-<20 |
| 111 | 11 | 0.8h | 40 | D34428 | 0.35 | B.5-<20 |
| 112 | 11 | 16.0h | 32 | D34428 | 0.25 | B.5-<20 |
| 113 | 11 | 8.16h | 40 | D34428 | 0.35 | B.5-<20 |
| 114 | 11 | 0.8h | 134 | D34433 | 0.238806 | B.5-<20 |
| 115 | 11 | 16.0h | 37 | D34433 | 0.135135 | B.5-<20 |
| 116 | 11 | 8.16h | 50 | D34433 | 0.08 | B.5-<20 |
| 117 | 11 | 0.8h | 24 | D34435 | 0.083333 | B.5-<20 |
| 118 | 11 | 16.0h | 16 | D34435 | 0 | B.5-<20 |
| 119 | 11 | 0.8h | 74 | D67389 | 0.662162 | B.5-<20 |
| 120 | 11 | 16.0h | 64 | D67389 | 0.171875 | B.5-<20 |
| 121 | 11 | 8.16h | 16 | D67389 | 0.5 | B.5-<20 |
| 122 | 11 | 0.8h | 8 | D67468 | 0.125 | B.5-<20 |
| 123 | 11 | 8.16h | 16 | D67468 | 0.1875 | B.5-<20 |
| 124 | 12 | 0.8h | 75 | D109776 | 0.44 | B.5-<20 |
| 125 | 12 | 16.0h | 24 | D109776 | 0.083333 | B.5-<20 |
| 126 | 12 | 8.16h | 8 | D109776 | 0.25 | B.5-<20 |
| 127 | 12 | 0.8h | 148 | D34428 | 0.452703 | B.5-<20 |
| 128 | 12 | 16.0h | 57 | D34428 | 0.333333 | B.5-<20 |
| 129 | 12 | 8.16h | 40 | D34428 | 0.325 | B.5-<20 |
| 130 | 12 | 0.8h | 536 | D34433 | 0.309701 | B.5-<20 |
| 131 | 12 | 16.0h | 371 | D34433 | 0.207547 | B.5-<20 |
| 132 | 12 | 8.16h | 301 | D34433 | 0.17608 | B.5-<20 |
| 133 | 12 | 0.8h | 58 | D34435 | 0.137931 | B.5-<20 |
| 134 | 12 | 16.0h | 119 | D34435 | 0.193277 | B.5-<20 |
| 135 | 12 | 8.16h | 31 | D34435 | 0.16129 | B.5-<20 |
| 136 | 12 | 0.8h | 247 | D67389 | 0.554656 | B.5-<20 |
| 137 | 12 | 16.0h | 72 | D67389 | 0.166667 | B.5-<20 |
| 138 | 12 | 8.16h | 37 | D67389 | 0.72973 | B.5-<20 |
| 139 | 12 | 0.8h | 8 | D67468 | 0.5 | B.5-<20 |
| 140 | 12 | 16.0h | 16 | D67468 | 0.5 | B.5-<20 |
| 141 | 12 | 8.16h | 8 | D67468 | 0.5 | B.5-<20 |
| 142 | 13 | 0.8h | 24 | D109776 | 0.375 | B.5-<20 |
| 143 | 13 | 16.0h | 85 | D109776 | 0.188235 | B.5-<20 |
| 144 | 13 | 8.16h | 16 | D109776 | 0.375 | B.5-<20 |
| 145 | 13 | 0.8h | 62 | D34428 | 0.370968 | B.5-<20 |
| 146 | 13 | 16.0h | 111 | D34428 | 0.324324 | B.5-<20 |
| 147 | 13 | 8.16h | 77 | D34428 | 0.233766 | B.5-<20 |
| 148 | 13 | 0.8h | 243 | D34433 | 0.27572 | B.5-<20 |
| 149 | 13 | 16.0h | 605 | D34433 | 0.247934 | B.5-<20 |
| 150 | 13 | 8.16h | 653 | D34433 | 0.17611 | B.5-<20 |
| 151 | 13 | 0.8h | 8 | D34435 | 0.125 | B.5-<20 |
| 152 | 13 | 16.0h | 58 | D34435 | 0.172414 | B.5-<20 |
| 153 | 13 | 8.16h | 66 | D34435 | 0.121212 | B.5-<20 |
| 154 | 13 | 0.8h | 216 | D67389 | 0.388889 | B.5-<20 |
| 155 | 13 | 16.0h | 214 | D67389 | 0.331776 | B.5-<20 |
| 156 | 13 | 8.16h | 170 | D67389 | 0.329412 | B.5-<20 |
| 157 | 13 | 8.16h | 16 | D67468 | 0.4375 | B.5-<20 |
| 158 | 14 | 0.8h | 16 | D109776 | 0.75 | B.5-<20 |
| 159 | 14 | 16.0h | 40 | D109776 | 0.225 | B.5-<20 |
| 160 | 14 | 8.16h | 49 | D109776 | 0.428571 | B.5-<20 |
| 161 | 14 | 0.8h | 33 | D34428 | 0.393939 | B.5-<20 |
| 162 | 14 | 16.0h | 56 | D34428 | 0.232143 | B.5-<20 |
| 163 | 14 | 8.16h | 128 | D34428 | 0.203125 | B.5-<20 |
| 164 | 14 | 0.8h | 71 | D34433 | 0.253521 | B.5-<20 |
| 165 | 14 | 16.0h | 288 | D34433 | 0.184028 | B.5-<20 |
| 166 | 14 | 8.16h | 273 | D34433 | 0.197802 | B.5-<20 |
| 167 | 14 | 0.8h | 27 | D34435 | 0.111111 | B.5-<20 |
| 168 | 14 | 16.0h | 8 | D34435 | 0.5 | B.5-<20 |
| 169 | 14 | 8.16h | 16 | D34435 | 0 | B.5-<20 |
| 170 | 14 | 0.8h | 181 | D67389 | 0.453039 | B.5-<20 |
| 171 | 14 | 16.0h | 263 | D67389 | 0.334601 | B.5-<20 |
| 172 | 14 | 8.16h | 121 | D67389 | 0.322314 | B.5-<20 |
| 173 | 14 | 16.0h | 16 | D67468 | 0.75 | B.5-<20 |
| 174 | 15 | 0.8h | 8 | D109776 | 1 | B.5-<20 |
| 175 | 15 | 0.8h | 16 | D34428 | 0.3125 | B.5-<20 |
| 176 | 15 | 16.0h | 46 | D34428 | 0.282609 | B.5-<20 |
| 177 | 15 | 8.16h | 64 | D34428 | 0.40625 | B.5-<20 |
| 178 | 15 | 0.8h | 64 | D34433 | 0.203125 | B.5-<20 |
| 179 | 15 | 8.16h | 52 | D34433 | 0.538462 | B.5-<20 |
| 180 | 15 | 8.16h | 8 | D34435 | 0.125 | B.5-<20 |
| 181 | 15 | 0.8h | 32 | D67389 | 0.5625 | B.5-<20 |
| 182 | 15 | 16.0h | 43 | D67389 | 0.372093 | B.5-<20 |
| 183 | 15 | 8.16h | 56 | D67389 | 0.25 | B.5-<20 |
| 184 | 16 | 16.0h | 8 | D34428 | 0.125 | B.5-<20 |
| 185 | 16 | 8.16h | 24 | D34428 | 0.25 | B.5-<20 |
| 187 | 16 | 8.16h | 8 | D34433 | 0.25 | B.5-<20 |
| 188 | 16 | 0.8h | 16 | D67389 | 1 | B.5-<20 |
| 189 | 16 | 16.0h | 22 | D67389 | 0.409091 | B.5-<20 |
| 190 | 17 | 0.8h | 8 | D34428 | 0.25 | B.5-<20 |
| 191 | 17 | 8.16h | 8 | D34428 | 0 | B.5-<20 |
| 192 | 17 | 8.16h | 24 | D34433 | 0.125 | B.5-<20 |
| 193 | 17 | 8.16h | 8 | D67389 | 0.125 | B.5-<20 |

## **ECI4**

File name “TS_ECI4.csv”

| ID | waterDepth | timeCat | prop1.5 | Animal | N | depthCat |
| --- | --- | --- | --- | --- | --- | --- |
| 2 | 1 | 0.8h | 0.881579 | D67389 | 76 | A.<5 |
| 3 | 1 | 16.0h | 0.625 | D67468 | 8 | A.<5 |
| 5 | 2 | 0.8h | 0.125 | D109776 | 16 | A.<5 |
| 6 | 2 | 0.8h | 0.714286 | D67468 | 14 | A.<5 |
| 7 | 2 | 0.8h | 0.844156 | D67389 | 154 | A.<5 |
| 9 | 2 | 0.8h | 0.125 | D34435 | 16 | A.<5 |
| 10 | 2 | 16.0h | 0.027778 | D109776 | 36 | A.<5 |
| 11 | 2 | 16.0h | 0.5 | D67468 | 18 | A.<5 |
| 12 | 2 | 16.0h | 0.693878 | D67389 | 147 | A.<5 |
| 13 | 2 | 16.0h | 0 | D34428 | 26 | A.<5 |
| 14 | 3 | 0.8h | 0.125 | D109776 | 16 | A.<5 |
| 15 | 3 | 0.8h | 0.54 | D67389 | 100 | A.<5 |
| 16 | 3 | 0.8h | 0 | D34428 | 18 | A.<5 |
| 17 | 3 | 0.8h | 0.008264 | D34435 | 121 | A.<5 |
| 18 | 3 | 16.0h | 0.1 | D109776 | 40 | A.<5 |
| 19 | 3 | 16.0h | 0.588235 | D67468 | 34 | A.<5 |
| 20 | 3 | 16.0h | 0.627329 | D67389 | 161 | A.<5 |
| 21 | 3 | 16.0h | 0 | D34428 | 8 | A.<5 |
| 22 | 3 | 16.0h | 0.086957 | D34435 | 46 | A.<5 |
| 23 | 4 | 0.8h | 0.1875 | D109776 | 16 | A.<5 |
| 24 | 4 | 0.8h | 0.46875 | D67389 | 64 | A.<5 |
| 25 | 4 | 0.8h | 0 | D34428 | 8 | A.<5 |
| 26 | 4 | 0.8h | 0.133333 | D34435 | 45 | A.<5 |
| 27 | 4 | 16.0h | 0.055556 | D109776 | 18 | A.<5 |
| 28 | 4 | 16.0h | 0.584416 | D67389 | 77 | A.<5 |
| 29 | 4 | 16.0h | 0 | D34428 | 10 | A.<5 |
| 30 | 4 | 16.0h | 0.071942 | D34435 | 139 | A.<5 |
| 31 | 4 | 8.16h | 0.125 | D67468 | 8 | A.<5 |
| 32 | 4 | 8.16h | 0.25 | D67389 | 8 | A.<5 |
| 33 | 4 | 8.16h | 0 | D34428 | 8 | A.<5 |
| 34 | 5 | 0.8h | 0.125 | D109776 | 16 | B.5-<20 |
| 36 | 5 | 0.8h | 0.25 | D67389 | 24 | B.5-<20 |
| 37 | 5 | 0.8h | 0.25 | D67468 | 8 | B.5-<20 |
| 38 | 5 | 0.8h | 0 | D34428 | 8 | B.5-<20 |
| 39 | 5 | 0.8h | 0 | D34435 | 42 | B.5-<20 |
| 40 | 5 | 16.0h | 0.142857 | D109776 | 14 | B.5-<20 |
| 41 | 5 | 16.0h | 0.222222 | D34433 | 9 | B.5-<20 |
| 42 | 5 | 16.0h | 0.538462 | D67389 | 39 | B.5-<20 |
| 43 | 5 | 16.0h | 0 | D34428 | 8 | B.5-<20 |
| 44 | 5 | 16.0h | 0.056818 | D34435 | 88 | B.5-<20 |
| 45 | 5 | 8.16h | 0.25 | D109776 | 8 | B.5-<20 |
| 46 | 5 | 8.16h | 0.375 | D67389 | 24 | B.5-<20 |
| 47 | 5 | 8.16h | 0.041667 | D34435 | 24 | B.5-<20 |
| 48 | 6 | 0.8h | 0.125 | D109776 | 16 | B.5-<20 |
| 49 | 6 | 0.8h | 0.125 | D34433 | 8 | B.5-<20 |
| 50 | 6 | 0.8h | 0.125 | D67389 | 16 | B.5-<20 |
| 51 | 6 | 0.8h | 0.125 | D67468 | 8 | B.5-<20 |
| 52 | 6 | 0.8h | 0 | D34428 | 16 | B.5-<20 |
| 53 | 6 | 0.8h | 0 | D34435 | 8 | B.5-<20 |
| 54 | 6 | 16.0h | 0 | D67389 | 8 | B.5-<20 |
| 55 | 6 | 16.0h | 0.1875 | D34433 | 32 | B.5-<20 |
| 56 | 6 | 16.0h | 0.083333 | D34435 | 24 | B.5-<20 |
| 57 | 6 | 8.16h | 0.090909 | D109776 | 22 | B.5-<20 |
| 58 | 6 | 8.16h | 0.25 | D34433 | 8 | B.5-<20 |
| 59 | 6 | 8.16h | 0.25 | D67389 | 24 | B.5-<20 |
| 60 | 6 | 8.16h | 0 | D34428 | 8 | B.5-<20 |
| 61 | 6 | 8.16h | 0 | D34435 | 40 | B.5-<20 |
| 62 | 7 | 0.8h | 0.0625 | D34433 | 16 | B.5-<20 |
| 63 | 7 | 0.8h | 0.25 | D67389 | 16 | B.5-<20 |
| 64 | 7 | 0.8h | 0 | D34428 | 8 | B.5-<20 |
| 65 | 7 | 0.8h | 0 | D34435 | 8 | B.5-<20 |
| 66 | 7 | 16.0h | 0.145833 | D34433 | 48 | B.5-<20 |
| 67 | 7 | 16.0h | 0.25 | D67389 | 16 | B.5-<20 |
| 68 | 7 | 16.0h | 0 | D34428 | 8 | B.5-<20 |
| 69 | 7 | 16.0h | 0.109375 | D34435 | 64 | B.5-<20 |
| 70 | 7 | 8.16h | 0.125 | D67389 | 8 | B.5-<20 |
| 71 | 7 | 8.16h | 0.148148 | D34433 | 27 | B.5-<20 |
| 72 | 7 | 8.16h | 0 | D34435 | 39 | B.5-<20 |
| 73 | 8 | 0.8h | 0.5 | D67389 | 8 | B.5-<20 |
| 74 | 8 | 0.8h | 0.025 | D34435 | 40 | B.5-<20 |
| 75 | 8 | 0.8h | 0.111111 | D34428 | 9 | B.5-<20 |
| 76 | 8 | 16.0h | 0.08 | D109776 | 25 | B.5-<20 |
| 77 | 8 | 16.0h | 0.25 | D67389 | 16 | B.5-<20 |
| 78 | 8 | 16.0h | 0 | D34428 | 8 | B.5-<20 |
| 79 | 8 | 16.0h | 0 | D34435 | 24 | B.5-<20 |
| 80 | 8 | 8.16h | 0 | D34428 | 8 | B.5-<20 |
| 81 | 8 | 8.16h | 0.071429 | D34435 | 14 | B.5-<20 |
| 82 | 9 | 0.8h | 0 | D34433 | 8 | B.5-<20 |
| 83 | 9 | 0.8h | 0.0625 | D109776 | 16 | B.5-<20 |
| 84 | 9 | 0.8h | 0.625 | D67389 | 8 | B.5-<20 |
| 85 | 9 | 0.8h | 0 | D34428 | 22 | B.5-<20 |
| 86 | 9 | 0.8h | 0.0625 | D34435 | 48 | B.5-<20 |
| 87 | 9 | 16.0h | 0.104167 | D109776 | 48 | B.5-<20 |
| 88 | 9 | 16.0h | 0.375 | D67389 | 8 | B.5-<20 |
| 90 | 9 | 16.0h | 0.142857 | D34435 | 56 | B.5-<20 |
| 91 | 9 | 8.16h | 0.125 | D109776 | 8 | B.5-<20 |
| 92 | 9 | 8.16h | 0 | D34435 | 8 | B.5-<20 |
| 93 | 9 | 8.16h | 0.125 | D34428 | 8 | B.5-<20 |
| 95 | 10 | 0.8h | 0.1 | D109776 | 10 | B.5-<20 |
| 96 | 10 | 0.8h | 0.25 | D67389 | 24 | B.5-<20 |
| 97 | 10 | 0.8h | 0 | D34428 | 15 | B.5-<20 |
| 98 | 10 | 0.8h | 0.041667 | D34435 | 24 | B.5-<20 |
| 99 | 10 | 16.0h | 0.030303 | D109776 | 33 | B.5-<20 |
| 100 | 10 | 16.0h | 0.461538 | D67468 | 13 | B.5-<20 |
| 101 | 10 | 16.0h | 0 | D34428 | 16 | B.5-<20 |
| 102 | 10 | 8.16h | 0.181818 | D67468 | 11 | B.5-<20 |
| 103 | 10 | 8.16h | 0.1875 | D34433 | 16 | B.5-<20 |
| 104 | 10 | 8.16h | 0.1875 | D67389 | 16 | B.5-<20 |
| 105 | 10 | 8.16h | 0.25 | D109776 | 8 | B.5-<20 |
| 106 | 10 | 8.16h | 0 | D34428 | 39 | B.5-<20 |
| 107 | 10 | 8.16h | 0.083333 | D34435 | 24 | B.5-<20 |
| 108 | 11 | 0.8h | 0.125 | D109776 | 72 | B.5-<20 |
| 109 | 11 | 0.8h | 0.125 | D67468 | 8 | B.5-<20 |
| 110 | 11 | 0.8h | 0.208955 | D34433 | 134 | B.5-<20 |
| 111 | 11 | 0.8h | 0.418919 | D67389 | 74 | B.5-<20 |
| 112 | 11 | 0.8h | 0 | D34435 | 24 | B.5-<20 |
| 113 | 11 | 0.8h | 0.125 | D34428 | 40 | B.5-<20 |
| 114 | 11 | 16.0h | 0.081081 | D34433 | 37 | B.5-<20 |
| 115 | 11 | 16.0h | 0.125 | D109776 | 8 | B.5-<20 |
| 116 | 11 | 16.0h | 0.15625 | D67389 | 64 | B.5-<20 |
| 117 | 11 | 16.0h | 0 | D34435 | 16 | B.5-<20 |
| 118 | 11 | 16.0h | 0.03125 | D34428 | 32 | B.5-<20 |
| 119 | 11 | 8.16h | 0 | D109776 | 8 | B.5-<20 |
| 120 | 11 | 8.16h | 0.04 | D34433 | 50 | B.5-<20 |
| 121 | 11 | 8.16h | 0.125 | D67468 | 16 | B.5-<20 |
| 122 | 11 | 8.16h | 0.25 | D67389 | 16 | B.5-<20 |
| 123 | 11 | 8.16h | 0.125 | D34428 | 40 | B.5-<20 |
| 124 | 12 | 0.8h | 0.093333 | D109776 | 75 | B.5-<20 |
| 125 | 12 | 0.8h | 0.238806 | D34433 | 536 | B.5-<20 |
| 126 | 12 | 0.8h | 0.331984 | D67389 | 247 | B.5-<20 |
| 127 | 12 | 0.8h | 0.375 | D67468 | 8 | B.5-<20 |
| 128 | 12 | 0.8h | 0.040541 | D34428 | 148 | B.5-<20 |
| 129 | 12 | 0.8h | 0.103448 | D34435 | 58 | B.5-<20 |
| 130 | 12 | 16.0h | 0.083333 | D109776 | 24 | B.5-<20 |
| 131 | 12 | 16.0h | 0.138889 | D67389 | 72 | B.5-<20 |
| 132 | 12 | 16.0h | 0.15903 | D34433 | 371 | B.5-<20 |
| 133 | 12 | 16.0h | 0.3125 | D67468 | 16 | B.5-<20 |
| 134 | 12 | 16.0h | 0.052632 | D34428 | 57 | B.5-<20 |
| 135 | 12 | 16.0h | 0.10084 | D34435 | 119 | B.5-<20 |
| 136 | 12 | 8.16h | 0.122924 | D34433 | 301 | B.5-<20 |
| 137 | 12 | 8.16h | 0.125 | D67468 | 8 | B.5-<20 |
| 138 | 12 | 8.16h | 0.25 | D109776 | 8 | B.5-<20 |
| 139 | 12 | 8.16h | 0.297297 | D67389 | 37 | B.5-<20 |
| 140 | 12 | 8.16h | 0.05 | D34428 | 40 | B.5-<20 |
| 141 | 12 | 8.16h | 0.16129 | D34435 | 31 | B.5-<20 |
| 142 | 13 | 0.8h | 0.125 | D109776 | 24 | B.5-<20 |
| 143 | 13 | 0.8h | 0.213992 | D34433 | 243 | B.5-<20 |
| 144 | 13 | 0.8h | 0.305556 | D67389 | 216 | B.5-<20 |
| 145 | 13 | 0.8h | 0 | D34435 | 8 | B.5-<20 |
| 146 | 13 | 0.8h | 0.032258 | D34428 | 62 | B.5-<20 |
| 147 | 13 | 16.0h | 0.129412 | D109776 | 85 | B.5-<20 |
| 148 | 13 | 16.0h | 0.2 | D34433 | 605 | B.5-<20 |
| 149 | 13 | 16.0h | 0.205607 | D67389 | 214 | B.5-<20 |
| 150 | 13 | 16.0h | 0.036036 | D34428 | 111 | B.5-<20 |
| 151 | 13 | 16.0h | 0.103448 | D34435 | 58 | B.5-<20 |
| 152 | 13 | 8.16h | 0.0625 | D109776 | 16 | B.5-<20 |
| 153 | 13 | 8.16h | 0.137825 | D34433 | 653 | B.5-<20 |
| 154 | 13 | 8.16h | 0.1875 | D67468 | 16 | B.5-<20 |
| 155 | 13 | 8.16h | 0.2 | D67389 | 170 | B.5-<20 |
| 156 | 13 | 8.16h | 0.051948 | D34428 | 77 | B.5-<20 |
| 157 | 13 | 8.16h | 0.075758 | D34435 | 66 | B.5-<20 |
| 158 | 14 | 0.8h | 0.125 | D109776 | 16 | B.5-<20 |
| 159 | 14 | 0.8h | 0.197183 | D34433 | 71 | B.5-<20 |
| 160 | 14 | 0.8h | 0.325967 | D67389 | 181 | B.5-<20 |
| 161 | 14 | 0.8h | 0.074074 | D34435 | 27 | B.5-<20 |
| 162 | 14 | 0.8h | 0.090909 | D34428 | 33 | B.5-<20 |
| 163 | 14 | 16.0h | 0.1 | D109776 | 40 | B.5-<20 |
| 164 | 14 | 16.0h | 0.15625 | D34433 | 288 | B.5-<20 |
| 165 | 14 | 16.0h | 0.231939 | D67389 | 263 | B.5-<20 |
| 166 | 14 | 16.0h | 0.3125 | D67468 | 16 | B.5-<20 |
| 167 | 14 | 16.0h | 0.053571 | D34428 | 56 | B.5-<20 |
| 168 | 14 | 16.0h | 0.375 | D34435 | 8 | B.5-<20 |
| 169 | 14 | 8.16h | 0.102041 | D109776 | 49 | B.5-<20 |
| 170 | 14 | 8.16h | 0.142857 | D34433 | 273 | B.5-<20 |
| 171 | 14 | 8.16h | 0.181818 | D67389 | 121 | B.5-<20 |
| 172 | 14 | 8.16h | 0 | D34435 | 16 | B.5-<20 |
| 173 | 14 | 8.16h | 0.03125 | D34428 | 128 | B.5-<20 |
| 174 | 15 | 0.8h | 0.1875 | D34433 | 64 | B.5-<20 |
| 175 | 15 | 0.8h | 0.25 | D67389 | 32 | B.5-<20 |
| 176 | 15 | 0.8h | 0.375 | D109776 | 8 | B.5-<20 |
| 177 | 15 | 0.8h | 0.125 | D34428 | 16 | B.5-<20 |
| 178 | 15 | 16.0h | 0.255814 | D67389 | 43 | B.5-<20 |
| 179 | 15 | 16.0h | 0.021739 | D34428 | 46 | B.5-<20 |
| 180 | 15 | 8.16h | 0.134615 | D34433 | 52 | B.5-<20 |
| 181 | 15 | 8.16h | 0.178571 | D67389 | 56 | B.5-<20 |
| 182 | 15 | 8.16h | 0 | D34435 | 8 | B.5-<20 |
| 183 | 15 | 8.16h | 0.0625 | D34428 | 64 | B.5-<20 |
| 184 | 16 | 0.8h | 0.8125 | D67389 | 16 | B.5-<20 |
| 185 | 16 | 16.0h | 0.363636 | D67389 | 22 | B.5-<20 |
| 187 | 16 | 16.0h | 0 | D34428 | 8 | B.5-<20 |
| 188 | 16 | 8.16h | 0.125 | D34433 | 8 | B.5-<20 |
| 189 | 16 | 8.16h | 0 | D34428 | 24 | B.5-<20 |
| 190 | 17 | 0.8h | 0 | D34428 | 8 | B.5-<20 |
| 191 | 17 | 8.16h | 0.125 | D34433 | 24 | B.5-<20 |
| 192 | 17 | 8.16h | 0.125 | D67389 | 8 | B.5-<20 |
| 193 | 17 | 8.16h | 0 | D34428 | 8 | B.5-<20 |

###########General linear mixed model for surface proportion

library(lme4) # version 0.999999-0

surface1.5 <- read.csv(file="TS_ECI4.csv") #Data for estimating availability bias for ECI4

head(surface1.5)

summary(surface1.5)

## Perform GLMM

prob1.5 <- glmer(prop1.5~depthCat+timeCat+ (1|Animal), family=binomial(link = "logit"),

data=surface1.5, weights=N/8)

summary(prob1.5)
